# ShenQiGan Extract Mitigates Intestinal Oxidative Stress in Weaned Piglets Through Bile Acid Metabolism Regulation and Gut Microbiota Remodeling via the Nrf2/Keap1 Pathway

**DOI:** 10.3390/antiox15101323

**Published:** 2026-10-14

**Authors:** Rongxia Guo, Chenghui Jiang, Xitong Yin, Chun Niu, Baoxia Chen, Ziwen Yuan, Hongjuan Wang, Yanming Wei, Yongli Hua

**Affiliations:** College of Veterinary Medicine, Gansu Agricultural University, Lanzhou 730070, China; 1073323010071@st.gsau.edu.cn (R.G.); 107332214003@st.gsau.edu.cn (C.J.); jiangchenghui1993@outlook.com (X.Y.); 1073323010070@st.gsau.edu.cn (C.N.); 1073323140019@st.gsau.edu.cn (B.C.); yuanzw@gsau.edu.cn (Z.Y.); 1073323140018@st.gsau.edu.cn (H.W.); weiym@gsau.edu.cn (Y.W.)

**Keywords:** ShenQiGan Extract, weaned piglets, gut microbiota, bile acid metabolism, Nrf2/Keap1, intestinal oxidative stress

## Abstract

Background: Weaning stress induces intestinal oxidative imbalance, barrier dysfunction, and gut microbial disturbances in piglets. ShenQiGan Extract (CAG), a traditional herbal-derived additive, has shown potential antioxidant and intestinal protective properties; however, the underlying mechanisms remain unclear. This study investigated whether CAG protects weaned piglets against oxidative stress through modulation of the gut microbiota–bile acid (BA) axis and antioxidant signaling pathways. Methods: Eighty 28-day-old weaned piglets were assigned to control, 0.1%, 0.5%, or 1.0% CAG supplementation groups for 28 days. Serum antioxidant parameters, intestinal and hepatic histomorphology, bile acid profiles in feces and serum, gut microbiota composition, and intestinal expression of BA metabolism- and Nrf2/Keap1-related molecules were analyzed. Furthermore, HDCA, 7-ketoLCA, and CDCA, identified as differential bile acids associated with CAG supplementation, were evaluated in an H_2_O_2_-induced oxidative stress model using IPEC-J2 cells. Results: CAG supplementation improved systemic antioxidant status, intestinal morphology, mucus barrier integrity, and hepatic histological damage in weaned piglets. CAG reshaped the gut microbial community by increasing microbial richness and Lactobacillus abundance, which was associated with alterations in BA profiles, particularly increased HDCA, 7-ketoLCA, and CDCA levels. CAG also regulated intestinal BA metabolism-related molecules, including BA synthesis, transport, and signaling pathways, and activated Nrf2/Keap1 antioxidant signaling. In vitro, HDCA, 7-ketoLCA, and CDCA alleviated H_2_O_2_-induced oxidative stress and improved epithelial barrier function, with CDCA showing the most consistent regulatory effects with the in vivo response. Conclusions: CAG supplementation protects weaned piglets against oxidative stress and intestinal barrier impairment by remodeling the gut microbiota–BA axis, regulating BA metabolism, and activating Nrf2/Keap1 antioxidant signaling. Among the identified differential BAs, CDCA may represent an important mediator contributing to the intestinal protective effects of CAG.

## 1. Introduction

Weaning represents a critical physiological stress period in commercial pig production [1,2,3]. Following weaning, multiple stressors, including abrupt dietary transition, environmental changes, and maternal separation, disrupt systemic redox homeostasis and induce excessive accumulation of reactive oxygen species (ROS) in the intestine. The resulting oxidative stress causes intestinal mucosal injury and compromises epithelial barrier integrity while simultaneously disturbing microbial colonization and bile acid homeostasis. These alterations collectively establish a vicious cycle of “oxidative damage-gut microbiota dysbiosis-metabolic disturbance”, ultimately leading to a high incidence of diarrhea, growth retardation, increased morbidity and mortality, and substantial economic losses to the swine industry [4,5,6].

In-feed antibiotics and pharmacological levels of zinc oxide have traditionally been used to alleviate intestinal injury associated with weaning stress. However, the long-term use of antibiotics promotes the emergence of antimicrobial-resistant bacteria and increases the risk of drug residues in animal-derived products, whereas the excretion of high-dose zinc oxide contributes to heavy-metal accumulation and soil contamination [7]. With the implementation of policies restricting or prohibiting the use of antibiotic growth promoters in animal feed, increasing attention has been directed toward the development of natural antibiotic alternatives with both antioxidant and microbiota-modulating properties. Conventional antioxidants generally act by directly scavenging free radicals but have limited capacity to regulate upstream microbiota-metabolite signaling networks. Consequently, they may be insufficient to fundamentally reverse persistent intestinal oxidative injury induced by weaning [8,9]. In contrast, natural bioactive extracts possess multiple biological activities, including antioxidative, microbiota-regulatory, and metabolic-modulatory effects, and therefore show considerable potential as alternatives to antibiotics. ShenQiGan Extract (CAG) is a traditional Chinese herbal formula composed of extracts from Astragalus membranaceus, Codonopsis pilosula, and Glycyrrhiza species. Previous studies have demonstrated that CAG enhances intestinal barrier function and modulates inflammatory responses and immunoglobulin levels [10,11,12]. As a plant-derived extract with potent antioxidant activity, CAG has considerable potential for development as a green feed additive and an alternative to antibiotics. Nevertheless, the integrated regulatory mechanisms by which CAG alleviates intestinal oxidative stress in weaned piglets remain to be systematically elucidated [13,14,15].

Weaning exposes piglets to a variety of stressors that can disturb intestinal redox balance. Excessive ROS production may elevate Keap1 levels, thereby facilitating ubiquitin-dependent proteasomal turnover of Nrf2 and restricting its entry into the nucleus. As a consequence, Nrf2-mediated antioxidant signaling is attenuated, leading to reduced transcription and protein abundance of several antioxidant effectors, such as heme oxygenase-1 (HO-1), NAD(P)H quinone dehydrogenase 1 (NQO1), superoxide dismutase (SOD), and glutathione peroxidase (GSH-Px). Collectively, these changes compromise the intrinsic antioxidant capacity of the intestine [16,17]. Prolonged oxidative damage can additionally impair the epithelial barrier by reducing tight-junction protein abundance and enhancing intestinal permeability, which may in turn contribute to disruption of the gut microbial community [18,19].

Bile acids originate from the host but are extensively modified by intestinal microorganisms, and these metabolites participate in signaling processes that contribute to intestinal physiological stability [20,21]. Oxidative stress can reshape the gut microbial community, characterized by a decline in commensal bacteria such as Lactobacillus and Faecalibacterium together with an expansion of potentially harmful taxa such as Streptococcus. This shift in microbial composition may reduce the availability of enzymes responsible for bile acid conversion, particularly bile salt hydrolase (BSH), ultimately altering microbial bile acid metabolism and the relative composition of primary versus secondary bile acids [22,23]. Beyond their metabolic roles, bile acids act as important mediators of intestinal signaling. Chenodeoxycholic acid (CDCA), a primary bile acid, together with secondary bile acids generated through microbial metabolism, can engage bile acid-responsive receptors, including the farnesoid X receptor/retinoid X receptor alpha (FXR/RXRα) axis and Takeda G protein-coupled receptor 5 (TGR5). Activation of these receptor-associated pathways may communicate with Nrf2-dependent antioxidant signaling, thereby contributing to protection against oxidative damage in the intestine [24,25].

Despite substantial progress, current studies investigating the mechanisms by which natural bioactive compounds regulate intestinal oxidative stress in weaned piglets remain subject to several limitations. First, most studies have independently examined alterations in either the gut microbiota or bile acid metabolism, without comprehensively investigating the intrinsic relationships among microbial composition, bile acid profiles, and antioxidant signaling pathways [26,27,28]. In the present study, we therefore proposed a “gut microbiota–bile acid metabolism-Nrf2/Keap1 antioxidant signaling” axis to systematically elucidate the underlying regulatory mechanism. Second, previous studies have predominantly relied on in vivo animal experiments, with limited validation at the intestinal epithelial cell level [24]. Furthermore, many studies have merely characterized changes in targeted bile acid profiles without evaluating whether the identified discriminatory bile acids directly regulate intestinal antioxidant capacity, mucosal barrier function, and key molecules involved in bile acid metabolism. Consequently, the specific bile acid species responsible for intestinal protection have not been definitively identified [29,30,31,32].

Accordingly, this study aimed to investigate whether CAG supplementation protects weaned piglets against intestinal oxidative stress through regulation of the gut microbiota–bile acid metabolism–Nrf2/Keap1 signaling axis. The protective effects of CAG were evaluated by assessing intestinal and hepatic morphology, antioxidant status, gut microbiota composition, bile acid profiles, and related molecular pathways. Furthermore, key differential bile acids identified in vivo were evaluated in an H_2_O_2_-induced IPEC-J2 oxidative stress model to determine their direct effects on epithelial antioxidant capacity, barrier function, and bile acid metabolism. This study provides new insights into the microbial and metabolic mechanisms underlying the intestinal protective effects of CAG and supports its potential application as an alternative feed additive.

## 2. Materials and Methods

### 2.1. Animal Experimental Design

Eighty 28-day-old weaned piglets (Large White × Landrace × Duroc; body weight 7.78 ± 0.59 kg) were randomly assigned to four groups: the control group (Control), low-dose group (LCAG; 0.1% CAG), medium-dose group (MCAG; 0.5% CAG), and high-dose group (HCAG; 1.0% CAG). The feeding trial lasted for 28 days. On day 29, blood samples were collected from the anterior vena cava of piglets in the Control and HCAG groups, and serum samples were obtained for antioxidant parameter analysis. Eight piglets from each of these two groups were randomly selected and euthanized. The middle jejunum, middle ileum, and liver tissues were collected. Portions of the tissues were fixed in 10% neutral buffered formalin for morphological analysis, while the remaining tissues were stored at −80 °C for subsequent analyses. Cecal contents were collected under sterile conditions and immediately stored at −80 °C. Detailed procedures for sample collection and CAG preparation, as well as analyses of the bioactive components of CAG and the nutrient composition of the basal diet, have been described in our previous study [33].

### 2.2. Phytochemical Characterization of ShenQiGan Extract

The phytochemical composition of ShenQiGan Extract (CAG) used in the present study was previously characterized using high-performance liquid chromatography (HPLC) according to our established analytical procedure. Briefly, chromatographic analysis was performed using an Agilent 1260 Infinity HPLC system equipped with a diode-array detector (Agilent Technologies, Santa Clara, CA, USA)and an Agilent ZORBAX SB-C18 analytical column (250 mm × 4.6 mm, 5 μm). The mobile phase consisted of 0.1% (*v*/*v*) aqueous phosphoric acid and chromatographic-grade acetonitrile under gradient elution.

As shown in Table 1, seven representative phytochemical constituents were identified and quantified, including calycosin-7-glucoside, lobetyolin, liquiritin, ononin, calycosin, liquiritigenin, and formononetin. Their concentrations were 19.8, 23.1, 86.8, 14.2, 16.4, 35.7, and 17.2 μg/mL, respectively. In addition, the total polysaccharide and flavonoid contents of CAG were previously determined to be 694.1 mg/g and 11.689 mg/g, respectively [33]. These phytochemical data were obtained during our previous characterization of CAG and are cited here to define the chemical profile of the extract used in the present study.

### 2.3. Scanning Electron Microscopy

Fresh ileal tissue was excised from the middle segment and cut into blocks of approximately 3 mm × 3 mm × 3 mm. The specimens were promptly placed in 2.5% glutaraldehyde for fixation. They were then prepared for scanning electron microscopic observation through sequential dehydration, drying, and gold sputter coating following routine processing procedures. The resulting specimens were visualized by scanning electron microscopy to characterize villus architecture, surface integrity, microvillus organization, and the ultrastructural features of the intestinal epithelial surface.

### 2.4. Hematoxylin and Eosin Staining

Fresh samples of the jejunum, ileum, and liver were obtained and preserved in 4% paraformaldehyde at room temperature for 48 h. After fixation, the tissues underwent dehydration and paraffin embedding, followed by section preparation and hematoxylin–eosin (H&E) staining. The stained sections were subsequently sealed with neutral resin and observed by light microscopy for histomorphological assessment.

### 2.5. Periodic Acid-Schiff Staining

Paraffin sections prepared from the jejunum and ileum were deparaffinized and rehydrated before periodic acid oxidation. The sections were subsequently incubated with Schiff reagent in the dark, followed by hematoxylin counterstaining, dehydration, clearing, and mounting. Goblet cells within the intestinal mucosa were then observed and quantified by light microscopy.

### 2.6. 16S rRNA Gene Sequencing Analysis of Fecal Microbiota

Fecal specimens were obtained for microbial DNA isolation. The V3–V4 hypervariable region of the bacterial 16S rRNA gene was amplified by PCR, after which sequencing libraries were prepared and subjected to high-throughput sequencing. The resulting sequence reads were analyzed with QIIME 2 (version 2019.4) to evaluate the taxonomic structure and diversity of the fecal microbiota.

### 2.7. Measurement of Serum Antioxidant Parameters

Serum GSH-Px, CAT, and SOD activities, together with the MDA concentration, were measured with commercially available assay kits from Nanjing Jiancheng Bioengineering Institute (Nanjing, China) following the protocols provided by the manufacturer.

### 2.8. Targeted Bile Acid Metabolomic Analysis of Fecal and Serum Samples

Serum and fecal samples were pretreated according to the requirements of the targeted metabolomic analysis. The target bile acid components were extracted and enriched, followed by targeted metabolomic analysis using a liquid chromatography–tandem mass spectrometry (LC-MS/MS) platform. The relative or absolute concentrations of individual bile acids were calculated using the corresponding calibration curves and subjected to statistical analysis.

### 2.9. Establishment of an Oxidative Stress Model in IPEC-J2 Cells and Screening of Optimal Bile Acid Concentrations

IPEC-J2 cells were initially propagated in T25 culture flasks, with 5 × 10^5^ cells placed in each flask containing 5 mL of complete growth medium. The culture medium was high glucose Dulbecco’s modified Eagle’s medium (DMEM; Biosharp, Hefei, China; BL304A) containing 10% fetal bovine serum (Biosharp, BL205A) and 1% penicillin-streptomycin (Solarbio, P7630). Cells were cultured at 37 °C in a humidified atmosphere containing 5% CO_2_. After two to three passages and confirmation of stable morphology and viability, the cells were transferred to 96-well plates at 1 × 10^4^ cells/well in a final volume of 100 μL. Following a 24 h attachment period, the cultures were incubated for 12 h with a range of concentrations of hyodeoxycholic acid (HDCA; Shanghai Zhenzhun Biotech, ZC-53335), 7-ketolithocholic acid (7-K-LCA; Shanghai Zhenzhun Biotech, ZS-20055), or chenodeoxycholic acid (CDCA; Shanghai Zhenzhun Biotech, ZC-57223). Thereafter, oxidative challenge was induced by incubation with graded concentrations of hydrogen peroxide (H_2_O_2_; Shandong Lierkang Biotech) for 4 h. Cell viability was subsequently assessed to determine suitable H_2_O_2_ conditions for inducing oxidative injury and to select the appropriate treatment concentration for each bile acid.

### 2.10. Cell Viability Assay

IPEC-J2 cells were seeded into 96-well plates at a density of 1 × 10^4^ cells per well and cultured for 24 h to allow cell attachment. When the cells reached approximately 80% confluence, they were subjected to the indicated treatments. Subsequently, CCK-8 reagent (Guangzhou Hucheng Biotech, HC0854) was added to each well, followed by incubation for 3 h. The absorbance at 450 nm was measured using a microplate reader (Thermo Scientific), and cell viability was calculated based on the optical density values.

### 2.11. Real-Time Quantitative Reverse Transcription Polymerase Chain Reaction (RT-qPCR)

Total RNA was obtained from tissue and cultured cell samples with TRIzol reagent (TransGen Biotech, ET101), following the protocol supplied by the manufacturer. The extracted RNA was then converted into complementary DNA (cDNA) with a reverse transcription kit (Accurate Biotech, AG11728). Subsequently, the synthesized cDNA served as the starting material for real-time quantitative PCR (qPCR) analysis of the genes of interest. GAPDH was included as the internal control for normalization. Detailed information on the primers employed for qPCR is listed in Table S1.

### 2.12. Western Blotting

Total proteins were extracted from jejunal tissues and IPEC-J2 cells for Western blot analysis. For animal samples, jejunal tissues from three biological replicates per group were randomly selected from the collected samples (*n* = 8 per group) for protein analysis. For cell experiments, Western blot analysis was performed using samples obtained from three independent experiments. Proteins were extracted from tissue and cell specimens with protein lysis buffer (Solarbio, R0010). Following centrifugation at 12,000× *g* for 10 min, the clarified supernatant was retained for subsequent analysis. Each protein preparation was combined with loading buffer (Solarbio, P1040) and heated at 100 °C for 10 min to achieve denaturation. For electrophoretic separation, 5 μL of each prepared sample was loaded onto a sodium dodecyl sulfate-polyacrylamide gel. Proteins resolved by electrophoresis were then electroblotted onto polyvinylidene fluoride (PVDF) membranes through wet transfer. Following electrophoresis, gels were sectioned according to the expected molecular-weight ranges of the target proteins before transfer to PVDF membranes. The corresponding gel regions were separately transferred onto PVDF membranes, while molecular-weight markers were retained to identify the target-protein positions. Target proteins and GAPDH were detected from membrane strips derived from the same electrophoretic gel and identical protein samples. Protein expression levels were normalized to the corresponding GAPDH signals. Before antibody probing, nonspecific binding sites on the membranes were saturated with 5% non-fat milk for 2 h. The membranes were subsequently exposed overnight to primary antibodies against GAPDH (ProteintechGroup, Wuhan, China; Cat. No.23019629), ZO-1 (Bioss Antibodies, Beijing, China; Cat. No.bs-1329R), occludin (Proteintech Group, Wuhan, China; Cat. No.13409-1-AP), claudin-1 (Bioss Antibodies, Beijing, China; Cat. No.CPA2349), CYP7A1 (Bioss Antibodies, Beijing, China; Cat. No.bs-21430R), CYP27A1 (Bioss Antibodies, Beijing, China; Cat. No. bs-5049R), CYP8B1 (Affinity Biosciences, Cincinnati, OH, USA; Cat. No. DF4762), ASBT (Bioss Antibodies, Beijing, China; Cat. No. bs-23146R), TGR5 (Boster Biological Technology, Wuhan, China; Cat. No.A01958-2), FXR (Bioss Antibodies, Beijing, China; Cat. No. bs-12867R), RXRα (Bioss Antibodies, Beijing, China; Cat. No. bs-20769R), ABCB11 (AffinityBiosciences, Cincinnati, OH, USA; Cat. No. AF7593), and FGF15 (CUSABIO Technology LLC, Houston, TX, USA; Cat. No. CSB-PA522052LA01MO). Following washing, the blots were reacted for 40 min with horseradish peroxidase-conjugated Affinipure goat anti-rabbit IgG (H + L) secondary antibody (ProteintechGroup, Wuhan, China; Cat. No. SA00001-2 SA00001-2). Chemiluminescent signals corresponding to the protein bands were finally captured with an Amersham ImageQuant 800 imaging system(Cytiva, Marlborough, MA, USA).

### 2.13. Statistical Analysis

Statistical analyses were conducted with IBM SPSS Statistics 26.0. The normality of data distribution was assessed using the Shapiro–Wilk test, and homogeneity of variance was evaluated using Levene’s test before performing parametric statistical analyses. An independent-samples Student’s *t*-test was applied when two groups were compared, whereas datasets containing more than two groups were evaluated by one-way analysis of variance (ANOVA), with Tukey’s honestly significant difference test used for subsequent pairwise comparisons. Results are expressed as mean ± standard error of the mean (SEM), and statistical significance was defined as *p* < 0.05.

## 3. Results

### 3.1. Effects of CAG Supplementation on Serum Antioxidant Parameters in Weaned Piglets

As summarized in Table 2, LCAG supplementation resulted in higher serum GSH-Px and SOD activities and a lower MDA concentration than those observed in the control group (*p* < 0.05). Although CAT activity showed an upward trend following LCAG treatment, this change was not statistically significant (*p* = 0.075). In the MCAG group, GSH-Px activity was elevated and MDA concentration was reduced (*p* < 0.05), while SOD and CAT activities remained comparable to those of the control group (*p* > 0.05). In contrast, HCAG enhanced the activities of all three antioxidant enzymes, including GSH-Px, SOD, and CAT, while simultaneously reducing serum MDA concentration (*p* < 0.05). When considered together with our previous findings [33], these observations suggest that HCAG produced the strongest overall antioxidant response. Accordingly, a dietary CAG inclusion level of 1.0% was used in the subsequent experiments.

### 3.2. Effects of CAG on the Morphological Structures of the Intestine and Liver in Weaned Piglets

**Figure 1:**
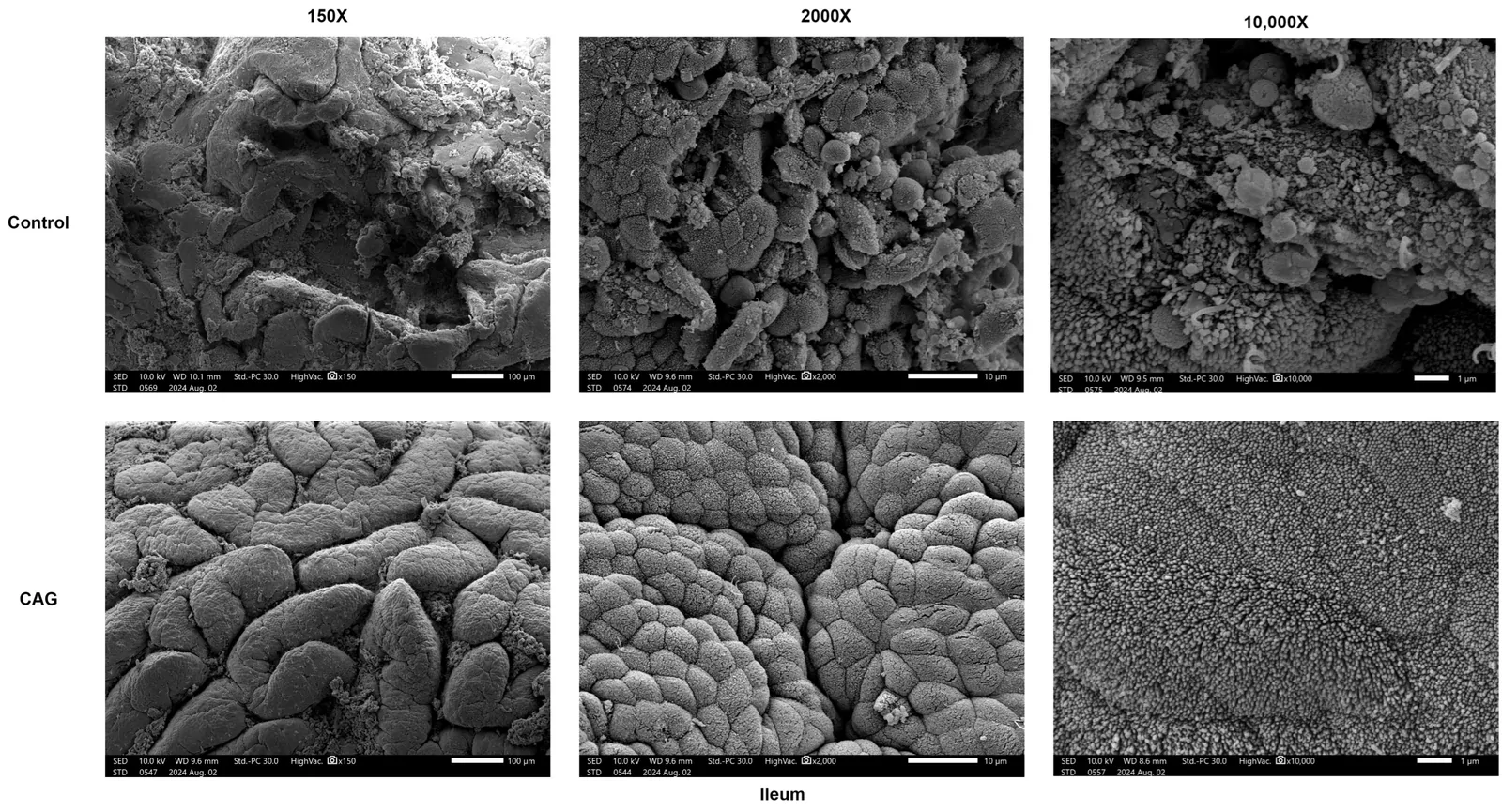
Scanning electron microscopy images of the ileum. Control, piglets fed the basal diet; CAG, piglets fed the basal diet supplemented with 1.0% CAG.

Scanning electron microscopy revealed that the ileal villi of piglets in the Control group exhibited irregular morphology and disorganized arrangement, with partial villous breakage and shedding. The epithelial cell boundaries were indistinct, and the microvilli were sparse and irregularly distributed. In contrast, the ileal villi in the CAG group were well organized and structurally intact, with a plump appearance. The epithelial cells were closely arranged, while the microvilli were dense and displayed a regular brush-border-like organization. These findings indicate that dietary CAG supplementation improved the ultrastructural integrity of the ileal mucosa and contributed to the maintenance of the intestinal epithelial barrier (Figure 1).

**Figure 2:**
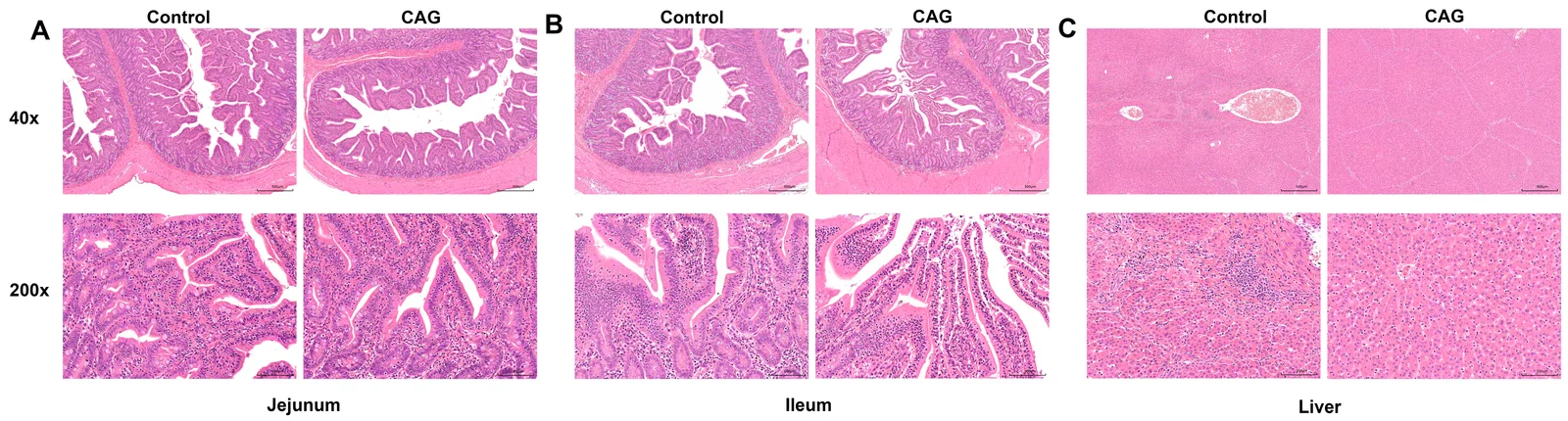
Hematoxylin and eosin staining of the jejunum, ileum, and liver. (**A**) Jejunum; (**B**) ileum; (**C**) liver. Control, piglets fed the basal diet; CAG, piglets fed the basal diet supplemented with 1.0% CAG.

As shown in Figure 2A,B, the jejunal and ileal mucosae of piglets in the Control group exhibited disorganized villous architecture, with partial villous atrophy, lodging, and shedding. The epithelial cells showed irregular morphology, the mucosal folds were reduced, and the overall integrity of the mucosal layer was compromised. By contrast, the CAG group displayed well-aligned and structurally intact villi with a plump appearance, closely arranged epithelial cells, abundant mucosal folds, and clearly distinguishable intestinal wall layers. In the liver, the Control group showed poorly defined hepatic lobular architecture, accompanied by mild hepatocellular vacuolar degeneration and swelling, dilation and congestion of the hepatic sinusoids, and evident inflammatory cell infiltration around the portal areas. In contrast, the CAG group exhibited well-preserved hepatic lobular architecture and regularly arranged hepatocytes, with no apparent cellular degeneration or necrosis. The hepatic sinusoidal spaces were generally normal, and inflammatory cell infiltration was markedly reduced (Figure 2C). Collectively, these observations suggest that CAG supplementation alleviated histopathological alterations in the jejunum, ileum, and liver of weaned piglets and helped preserve tissue structural integrity.

**Figure 3:**
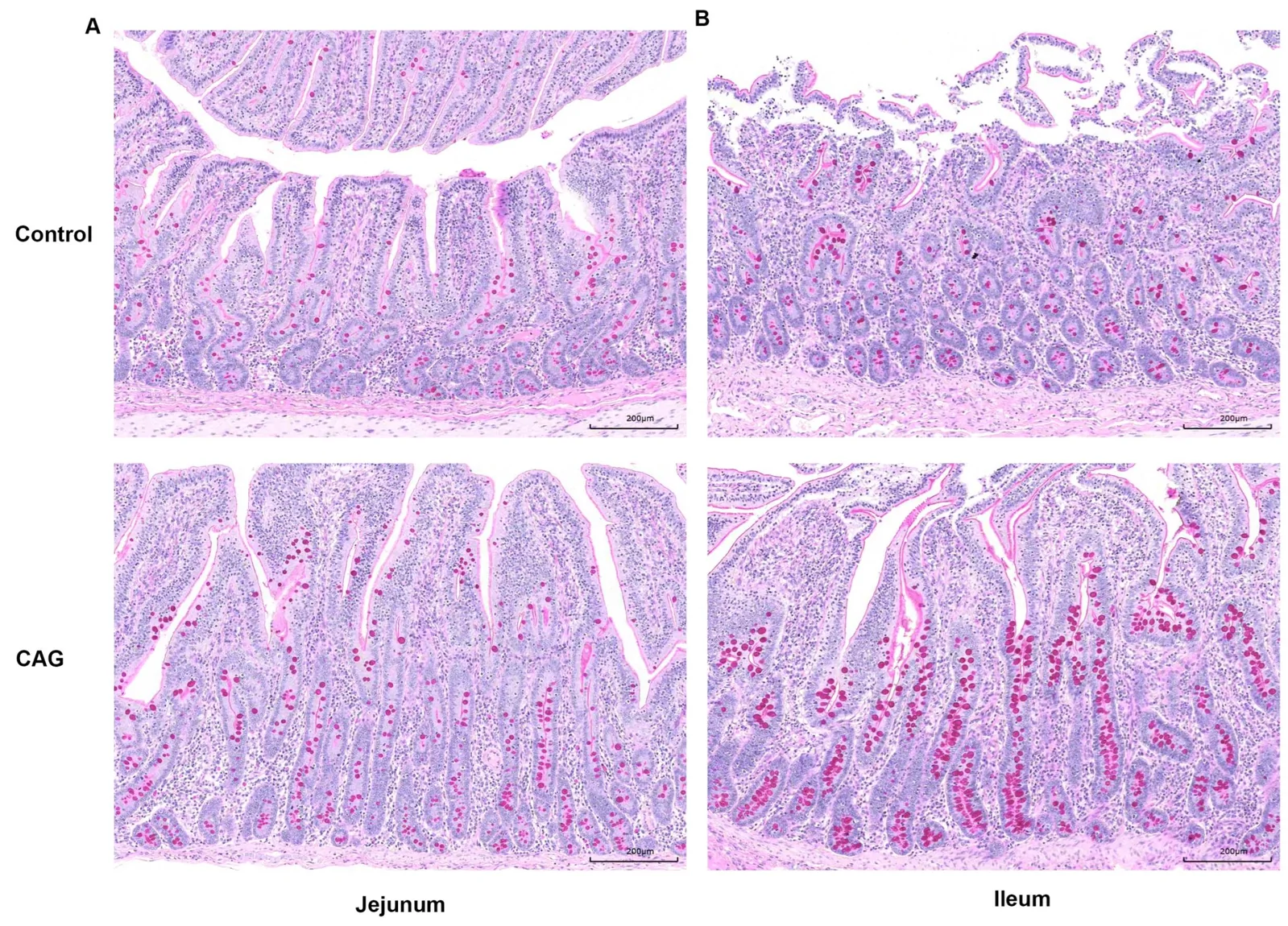
Periodic acid-Schiff staining of the jejunum and ileum. (**A**) Jejunum; (**B**) ileum. Control, piglets fed the basal diet; CAG, piglets fed the basal diet supplemented with 1.0% CAG.

As shown in Figure 3, the jejunum of piglets in the Control group contained relatively few goblet cells, with weak and unevenly distributed periodic acid-Schiff-positive signals and an indistinct mucus layer on the mucosal surface. In contrast, the CAG group showed an increased abundance of goblet cells, whose cytoplasm contained numerous magenta-stained mucin granules. Strong PAS-positive signals were continuously distributed along the longitudinal axis of the villi, and the mucus layer covering the mucosal surface appeared thicker. In the ileum, goblet cells in the Control group were sparsely distributed and were predominantly localized within the crypt regions. Only weak PAS-positive staining was observed in the villous epithelium, indicating limited mucus production. In comparison, the CAG group exhibited a greater abundance of goblet cells and intense PAS-positive staining in both the crypts and villous epithelium. Dense mucin granules and a relatively continuous mucus layer were also observed along the mucosal surface. These results suggest that CAG supplementation increased goblet cell abundance and mucus production in the jejunum and ileum, thereby enhancing the intestinal mucus barrier in weaned piglets.

Collectively, these observations suggest that CAG supplementation alleviated histopathological alterations in the jejunum, ileum, and liver of weaned piglets and helped preserve tissue structural integrity. Given the improvement in intestinal integrity and antioxidant status, we next investigated potential metabolic mechanisms underlying the protective effects of CAG, focusing on bile acid remodeling and related microbial alterations.

### 3.3. Effects of CAG Supplementation on the Fecal Bile Acid Profile of Weaned Piglets

**Figure 4:**
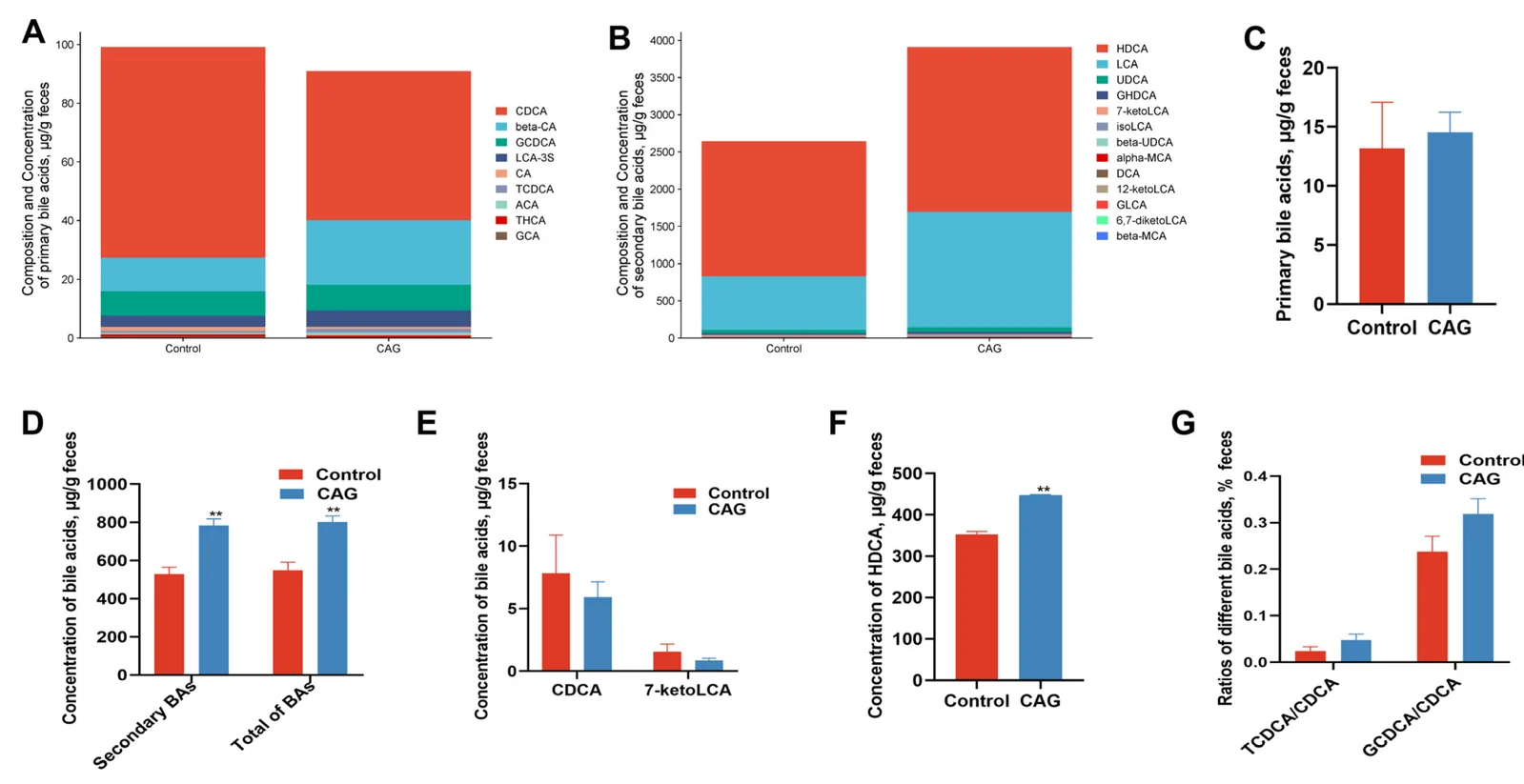
Effects of CAG supplementation on the fecal bile acid (BA) profile of weaned piglets (*n* = 5). (**A**) Composition of primary bile acids in the feces of weaned piglets. (**B**) Composition of secondary bile acids in the feces of weaned piglets. (**C**) Comparison of fecal primary bile acid concentrations between the Control and CAG groups. (**D**) Comparison of fecal secondary bile acid and total bile acid concentrations between the Control and CAG groups. (**E**) Comparison of fecal CDCA and 7-ketoLCA concentrations between the Control and CAG groups. (**F**) Comparison of fecal HDCA concentrations between the Control and CAG groups. (**G**) Ratios of different bile acids in the Control and CAG groups. Control, piglets fed the basal diet; CAG, piglets fed the basal diet supplemented with 1.0% CAG extract. ** *p* < 0.01 compared with the Control group.

Among the bile acids identified in the feces of weaned piglets, the major primary bile acids included chenodeoxycholic acid (CDCA), β-cholic acid (β-CA), glycochenodeoxycholic acid (GCDCA), lithocholic acid-3-sulfate (LCA-3S), and cholic acid (CA) (Figure 4A). The major secondary bile acids consisted of hyodeoxycholic acid (HDCA), lithocholic acid (LCA), ursodeoxycholic acid (UDCA), glycohyodeoxycholic acid (GHDCA), and 7-ketolithocholic acid (7-ketoLCA) (Figure 4B). According to Figure 4C–F, CAG supplementation did not alter the fecal level of primary bile acids relative to the Control group (*p* = 0.75), whereas the levels of secondary and total bile acids were higher in the CAG group (*p* < 0.01). Fecal CDCA and 7-ketoLCA concentrations were comparable between the two groups (*p* > 0.05), while HDCA was markedly higher following CAG supplementation (*p* < 0.001). Moreover, no between-group differences were detected in the TCDCA/CDCA or GCDCA/CDCA ratios (*p* > 0.05; Figure 4G).

### 3.4. Effects of CAG Supplementation on the Serum Bile Acid Profile of Weaned Piglets

**Figure 5:**
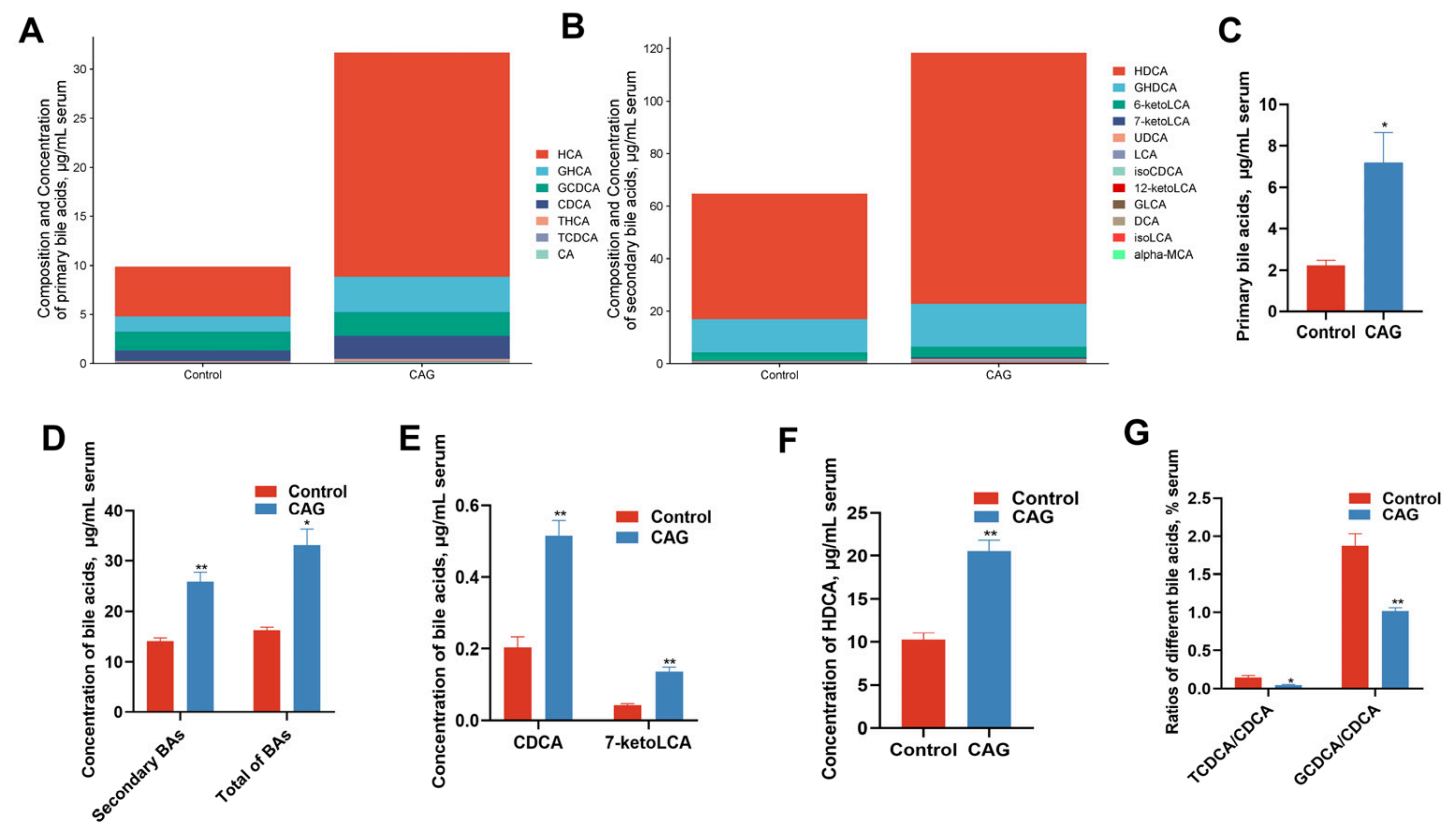
Effects of CAG supplementation on the serum bile acid profile of weaned piglets (*n* = 5). (**A**) Composition of primary bile acids in the serum of weaned piglets. (**B**) Composition of secondary bile acids in the serum of weaned piglets. (**C**) Comparison of serum primary bile acid concentrations between the Control and CAG groups. (**D**) Comparison of serum secondary bile acid and total bile acid concentrations between the Control and CAG groups. (**E**) Comparison of serum CDCA and 7-ketoLCA concentrations between the Control and CAG groups. (**F**) Comparison of serum HDCA concentrations between the Control and CAG groups. (**G**) Ratios of selected bile acids in the Control and CAG groups. Control, piglets fed the basal diet; CAG, piglets fed the basal diet supplemented with 1.0% CAG. * *p* < 0.05 and ** *p* < 0.01 compared with the Control group.

Figure 5A shows that the major primary bile acids present in the serum of weaned piglets included hyocholic acid (HCA), glycohyocholic acid (GHCA), glycochenodeoxycholic acid (GCDCA), chenodeoxycholic acid (CDCA), and taurohyocholic acid (THCA). The principal secondary bile acids comprised hyodeoxycholic acid (HDCA), glycohyodeoxycholic acid (GHDCA), 6-ketolithocholic acid (6-ketoLCA), 7-ketolithocholic acid (7-ketoLCA), and ursodeoxycholic acid (UDCA) (Figure 5B). According to Figure 5C–F, CAG supplementation resulted in higher serum levels of primary, secondary, and total bile acids relative to the Control group (*p* < 0.05). In addition, serum CDCA, 7-ketoLCA, and HDCA levels were markedly increased following CAG supplementation (*p* < 0.01). As illustrated in Figure 5G, both the TCDCA/CDCA and GCDCA/CDCA ratios were reduced in the CAG group compared with the Control group (*p* < 0.05). Collectively, these results suggest that CAG supplementation modified the circulating bile acid composition and may have facilitated bile acid deconjugation in weaned piglets.

Collectively, these results suggest that CAG supplementation modified the circulating bile acid composition and may have facilitated bile acid transformation in weaned piglets. Because intestinal microorganisms are major regulators of bile acid transformation, we further examined whether CAG-induced alterations in bile acid profiles were associated with changes in gut microbial composition.

### 3.5. Effects of CAG Supplementation on the Composition and Diversity of the Gut Microbiota in Weaned Piglets

**Figure 6:**
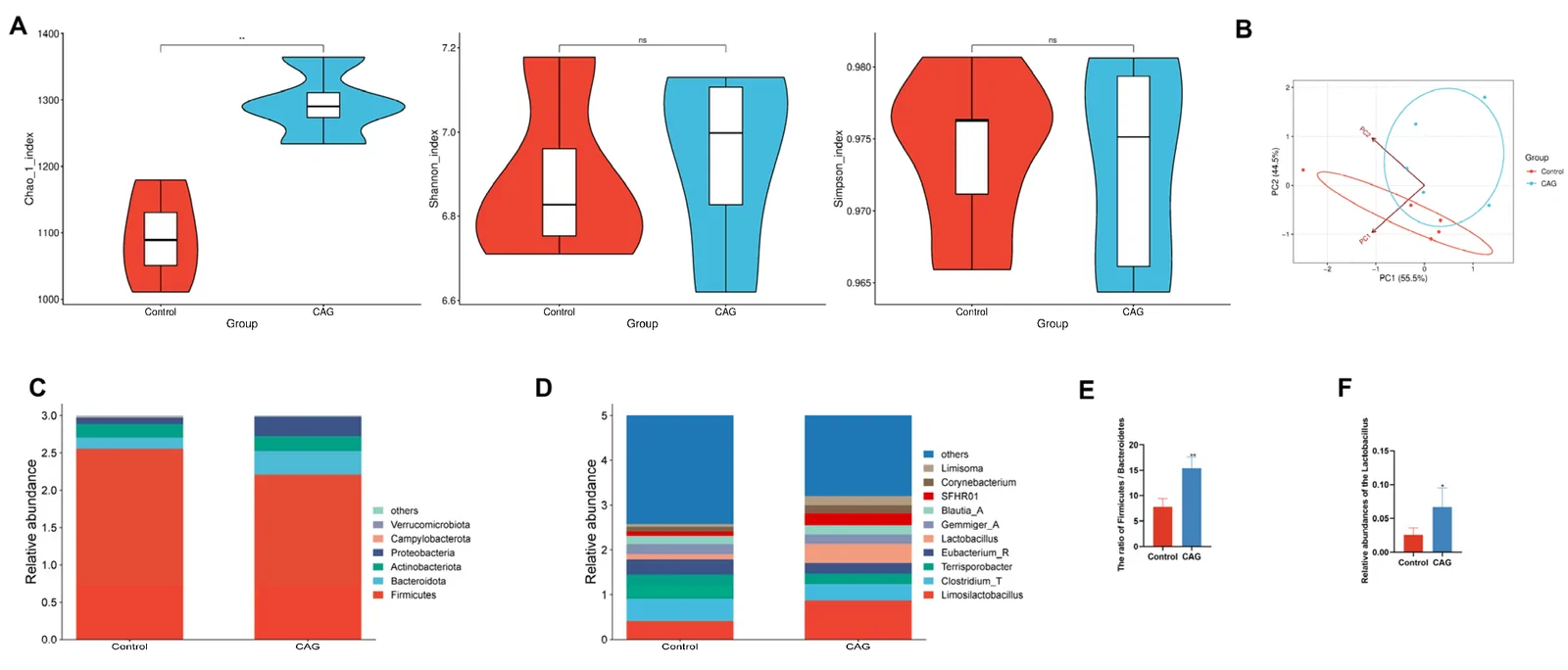
Effects of CAG supplementation on the composition and diversity of the gut microbiota in weaned piglets (*n* = 5). (**A**) Comparison of gut microbial α-diversity indices between the Control and CAG groups. (**B**) Principal coordinates analysis (PCoA) of gut microbial communities based on Bray–Curtis distances. (**C**) Relative abundances of bacterial taxa at the phylum level in weaned piglets from the Control and CAG groups. (**D**) Relative abundances of bacterial taxa at the genus level in weaned piglets from the Control and CAG groups. (**E**) *Firmicutes*-to-*Bacteroidota* ratio in weaned piglets from the Control and CAG groups. (**F**) Comparison of the relative abundance of *Lactobacillus* in weaned piglets between the Control and CAG groups. Control, piglets fed the basal diet; CAG, piglets fed the basal diet supplemented with 1.0% CAG. * *p* < 0.05 and ** *p* < 0.01 compared with the Control group.ns indicates no significant difference.The arrows indicate the direction and contribution of the corresponding variables to the separation of samples in the PCoA plot.

Figure 6A shows that CAG supplementation resulted in a higher Chao1 index than that observed in the Control group (*p* < 0.01), reflecting greater gut microbial species richness. By contrast, the Shannon and Simpson indices remained comparable between groups (*p* > 0.05), indicating that overall microbial diversity was largely unchanged. Bray–Curtis-based principal coordinates analysis (PCoA) separated the microbial communities of the Control and CAG groups into distinct clusters (Figure 6B), suggesting differences in their community structure. At the phylum level, the bacterial community was dominated by *Firmicutes*, *Bacteroidota*, *Actinobacteriota*, *Proteobacteria*, and *Campylobacterota*, which together represented more than 90% of the total bacterial population (Figure 6C). At the genus level, *Limosilactobacillus*, *Clostridium_T*, *Terrisporobacter*, *Eubacterium_R*, and *Lactobacillus* were the principal genera detected (Figure 6D). As illustrated in Figure 6E,F, the *Firmicutes*-to-*Bacteroidota* ratio was higher following CAG supplementation than in the Control group (*p* = 0.008). Likewise, Lactobacillus showed a greater relative abundance in the CAG group (*p* = 0.03).

### 3.6. Correlation Analysis Between Bile Acid Profiles and the Gut Microbiota

**Figure 7:**
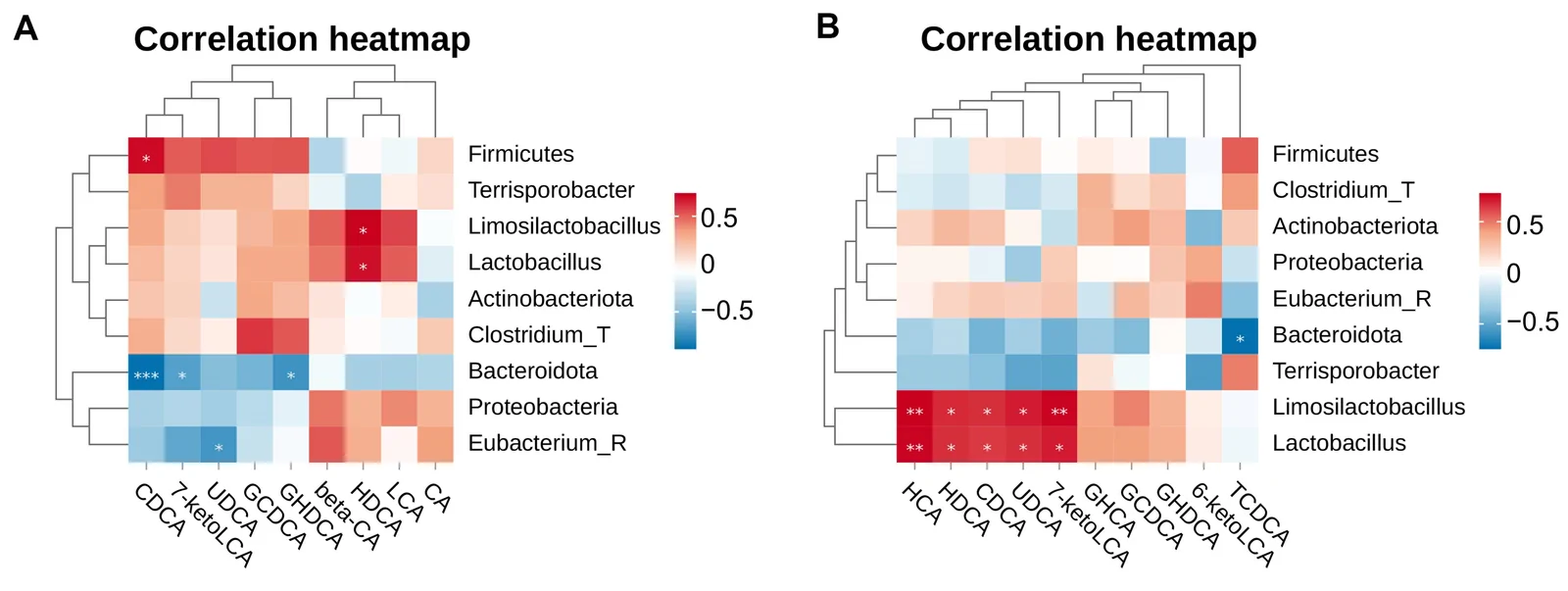
Spearman’s correlation analysis between bile acid profiles and the gut microbiota. (**A**) Correlations between the fecal bile acid profile and the gut microbiota in weaned piglets; (**B**) correlations between the serum bile acid profile and the gut microbiota in weaned piglets. * *p* < 0.05, ** *p* < 0.01, *** *p* < 0.001.

Spearman correlation analysis was used to assess the relationships between bile acid (BA) profiles and gut microbial composition. In fecal samples, HDCA showed strong positive associations with *Limosilactobacillus* and *Lactobacillus* (*p* < 0.05), while CDCA was strongly associated with *Firmicutes* in a positive manner (*p* < 0.05; Figure 7A). For serum BAs, HDCA, CDCA, and 7-ketolithocholic acid (7-ketoLCA) were all positively associated with *Limosilactobacillus* and *Lactobacillus* (*p* < 0.05; Figure 7B).

These findings suggest that CAG-associated enrichment of beneficial microbial taxa may contribute to the generation of bioactive bile acids, which may subsequently participate in regulating host antioxidant responses.

### 3.7. Effects of CAG on the Nrf2/Keap1 Oxidative Stress Signaling Pathway in Weaned Piglets

**Figure 8:**
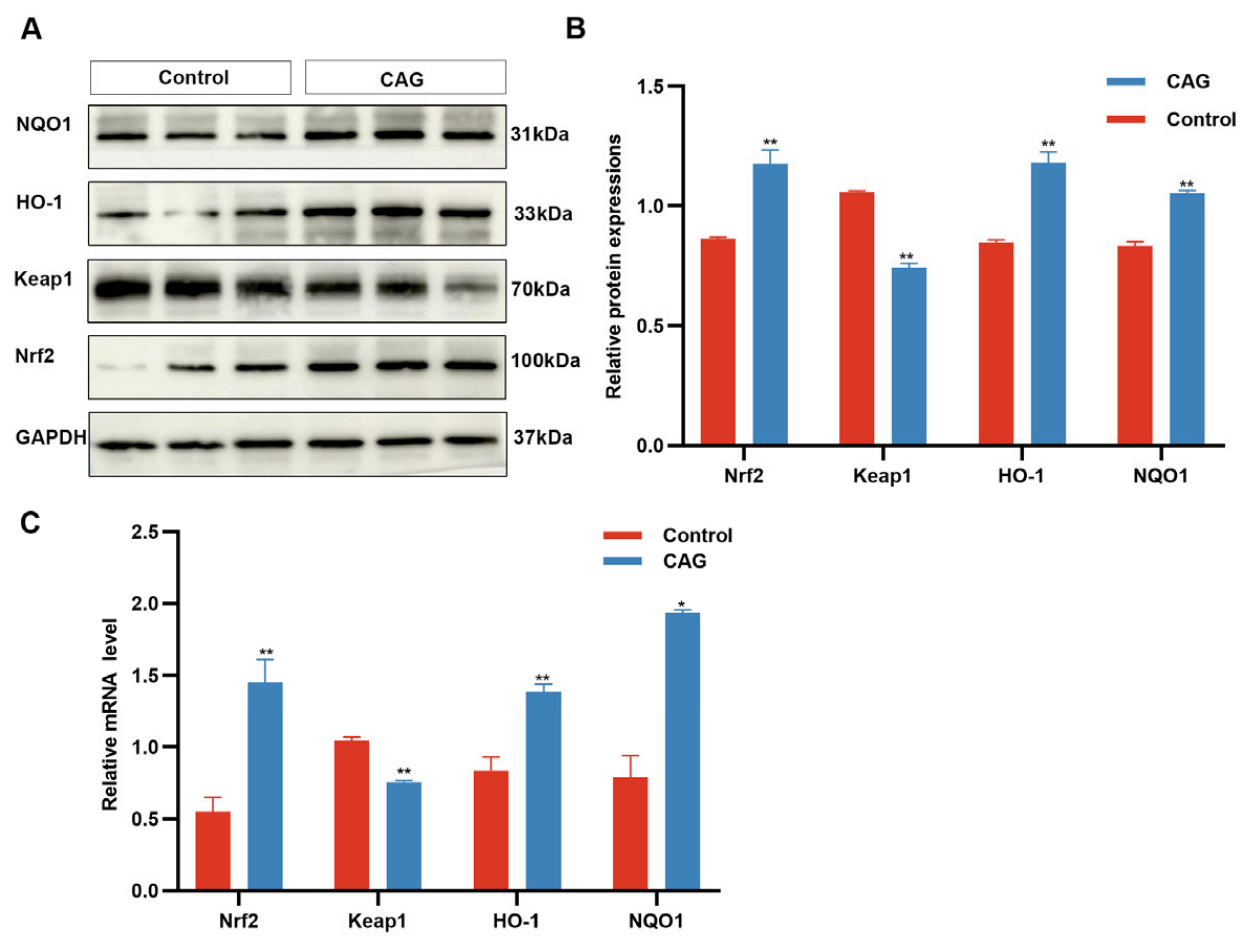
Effects of CAG on the intestinal Nrf2/Keap1 signaling pathway (*n* = 3). (**A**,**B**) Protein expression levels of Nrf2, Keap1, HO-1, and NQO1 in jejunal tissues. (**C**) Relative mRNA expression levels of Nrf2, Keap1, HO-1, and NQO1 in jejunal tissues. Data are presented as the mean ± SEM. * *p* < 0.05 and ** *p* < 0.01 compared with the Control group.

To elucidate the molecular mechanism by which CAG alleviates oxidative stress, the protein and gene expression levels of key components involved in the Nrf2/Keap1 signaling pathway were examined. As shown in Figure 8A,B, compared with the Control group, the CAG group exhibited significantly increased protein expression levels of Nrf2, HO-1, and NQO1 (*p* < 0.01), whereas the protein expression level of Keap1 was significantly decreased (*p* < 0.01). Consistently, the relative mRNA expression levels of Nrf2, HO-1, NQO1, and Keap1 showed expression patterns similar to those observed at the protein level (Figure 8C).

### 3.8. Effects of CAG on the Gene and Protein Expression of Key Molecules Involved in Bile Acid Metabolism in Weaned Piglets

**Figure 9:**
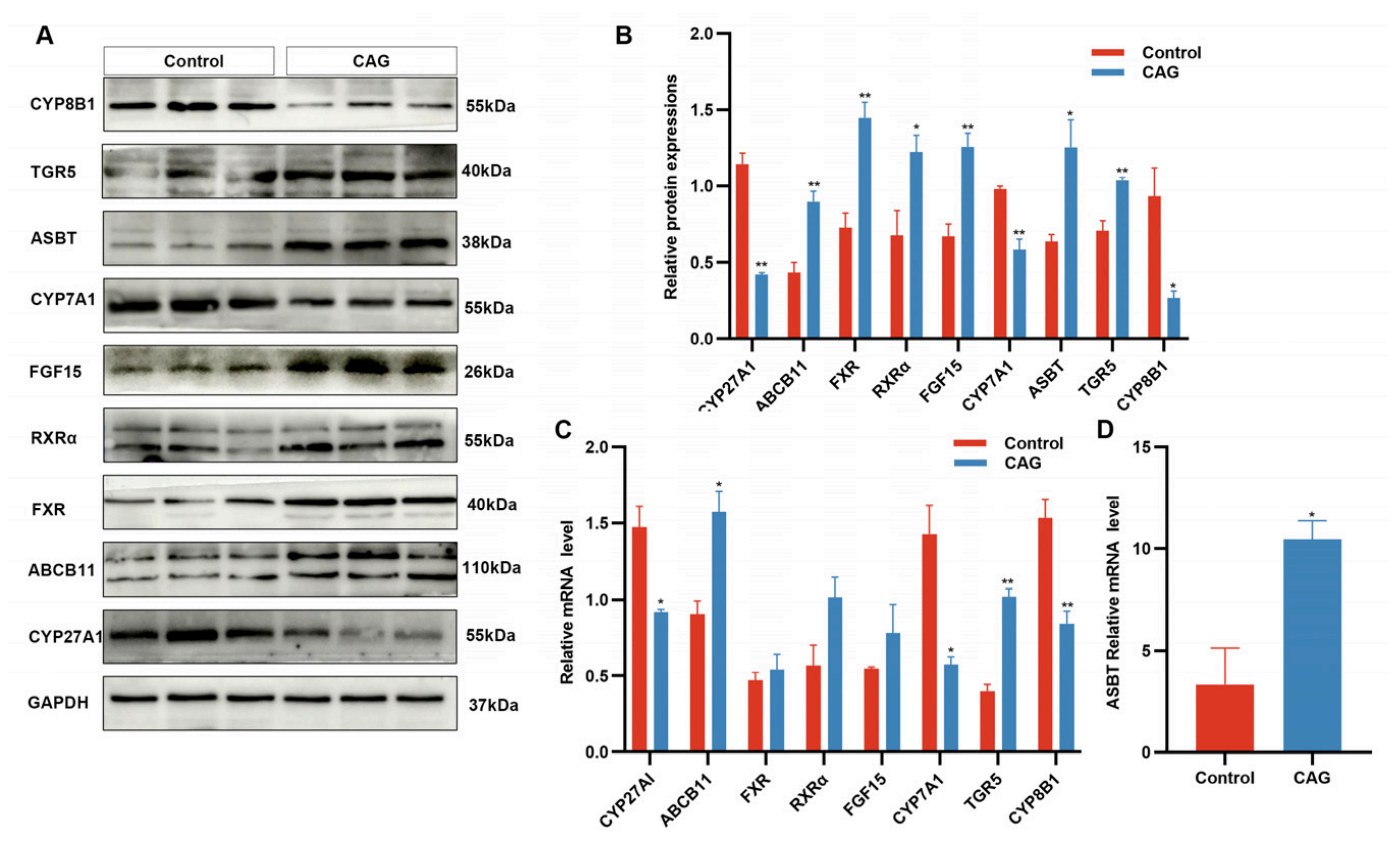
Expression profiles of bile acid metabolism-related molecules after CAG supplementation in weaned piglets(*n* = 3). (**A**,**B**) Protein expression levels of key molecules involved in bile acid metabolism. (**C**,**D**) Relative mRNA expression levels of key molecules involved in bile acid metabolism. Because ABCB11 mRNA was strongly upregulated, it is shown separately in (**D**). * *p* < 0.05 and ** *p* < 0.01 compared with the Control group.

Our previous findings showed that CAG markedly modified the profiles of selected bile acids in both serum and feces. To investigate the mechanisms responsible for CAG-induced changes in bile acid metabolism, we subsequently assessed key proteins and genes associated with bile acid metabolic pathways in jejunal tissues. This analysis was designed to determine whether the observed shifts in bile acid profiles were associated with intestinal signaling pathways governing bile acid metabolism. As presented in Figure 9A,B, CAG supplementation reduced the protein abundance of the bile acid-synthesizing enzymes CYP27A1, CYP7A1, and CYP8B1 relative to the Control group (*p* < 0.01). By contrast, ASBT and ABCB11, together with the nuclear receptors FXR and RXRα, showed higher protein abundance following CAG supplementation (*p* < 0.05). Protein levels of the membrane receptor TGR5 and the feedback regulator FGF15 were also elevated in the CAG group (*p* < 0.01). Similar changes were detected at the transcriptional level, with the mRNA profiles of these bile acid metabolism-related molecules generally paralleling their corresponding protein patterns (Figure 9C,D).

Based on the identification of HDCA, 7-ketoLCA, and CDCA as major differential bile acids associated with CAG supplementation, these metabolites were further evaluated in an intestinal epithelial cell model to determine their direct protective effects.

### 3.9. Effects of HDCA, 7-ketoLCA, and CDCA on H_2_O_2_-Induced Oxidative Stress in IPEC-J2 Cells

The preceding in vivo experiments showed that CAG markedly increased the abundance of three differential bile acids, HDCA, 7-ketoLCA, and CDCA, while enhancing Nrf2/Keap1 antioxidant signaling. To examine whether these bile acids contribute directly to the intestinal protective action of CAG, IPEC-J2 cells were employed to assess the effects of HDCA, 7-ketoLCA, and CDCA on cellular tolerance to oxidative stress.

A CCK-8 assay was initially conducted to establish suitable treatment concentrations for the three bile acids. Prior to bile acid exposure, the H_2_O_2_ concentration required to induce oxidative stress was optimized. As presented in Table 3, IPEC-J2 cells were exposed to 200, 400, 600, 800, 1000, 1200, or 1500 μmol/L H_2_O_2_ for 4 h, yielding cell viabilities of 97.82%, 92.73%, 77.78%, 68.16%, 64.77%, 62.93%, and 11.42%, respectively. Compared with the Control group (0 μmol/L H_2_O_2_), a significant decline in cell viability was observed at 600 μmol/L H_2_O_2_ (*p* < 0.001). Accordingly, 600 μmol/L H_2_O_2_ was chosen as the working concentration for inducing oxidative stress in IPEC-J2 cells. Because HDCA, 7-ketoLCA, and CDCA were prepared in dimethyl sulfoxide (DMSO), the potential influence of DMSO on cell viability was also assessed. DMSO at concentrations below 1.00% did not alter IPEC-J2 cell viability (*p* = 0.662; Table S2).

**Figure 10:**
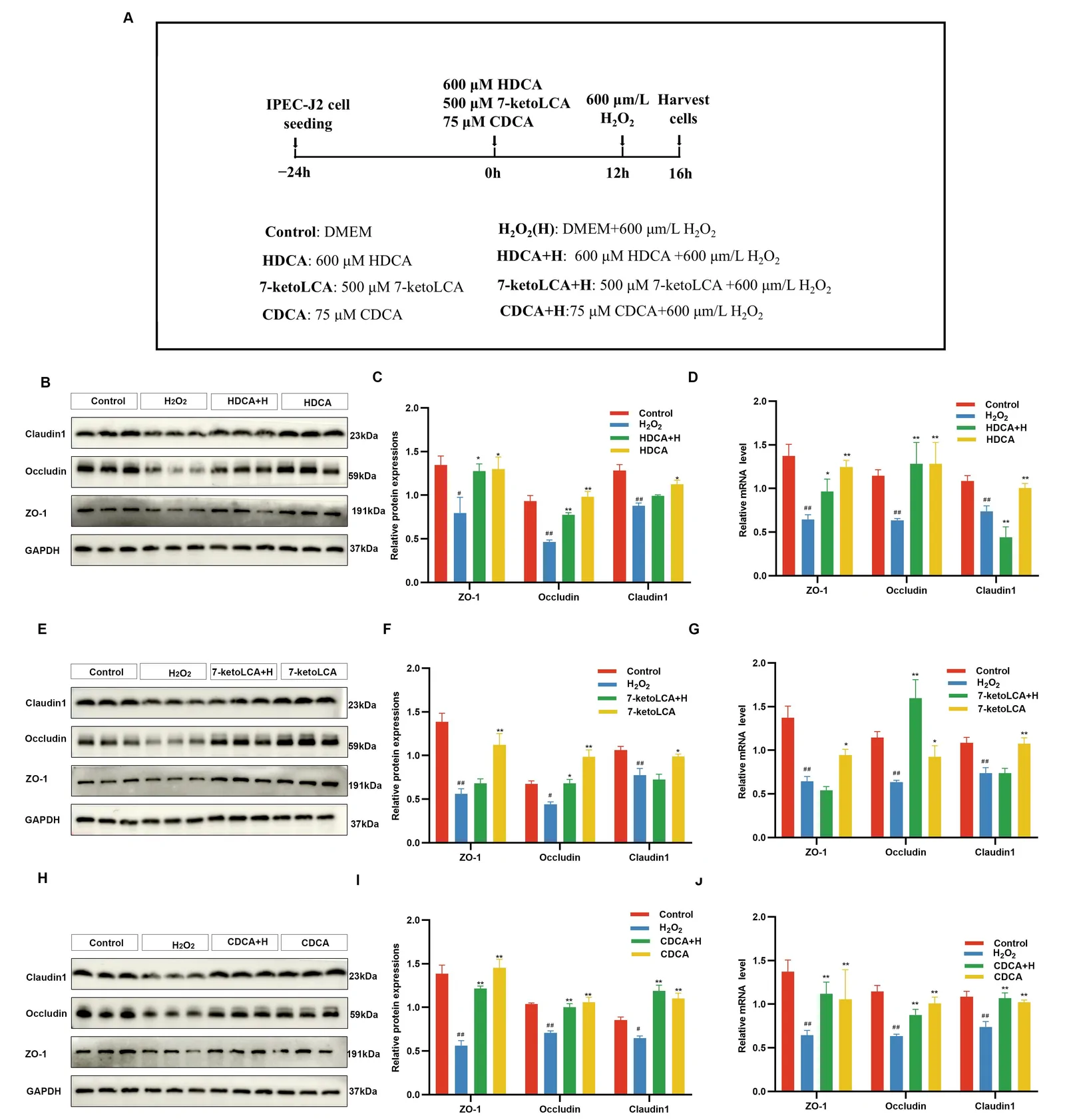
Effects of HDCA, 7-ketoLCA, and CDCA on the expression of barrier-related proteins and genes in IPEC-J2 cells (*n* = 3). (**A**) Schematic illustration of the experimental design. After seeding, the cells were allowed to adhere for 24 h and were subsequently pretreated with 600 μM HDCA, 500 μM 7-ketoLCA, or 75 μM CDCA for 12 h, followed by exposure to 600 μM H_2_O_2_ for 4 h. (**B**,**C**) Effects of 600 μM HDCA on the protein expression levels of barrier-related molecules in IPEC-J2 cells. (**D**) Effects of 600 μM HDCA on the mRNA expression levels of barrier-related molecules in IPEC-J2 cells. (**E**,**F**) Effects of 500 μM 7-ketoLCA on the protein expression levels of barrier-related molecules in IPEC-J2 cells. (**G**) Effects of 500 μM 7-ketoLCA on the mRNA expression levels of barrier-related molecules in IPEC-J2 cells. (**H**,**I**) Effects of 75 μM CDCA on the protein expression levels of barrier-related molecules in IPEC-J2 cells. (**J**) Effects of 75 μM CDCA on the mRNA expression levels of barrier-related molecules in IPEC-J2 cells. DMEM, Dulbecco’s modified Eagle’s medium. # *p* < 0.05 and ## *p* < 0.01 compared with the Control group; * *p* < 0.05 and ** *p* < 0.01 compared with the H_2_O_2_ group.

HDCA concentrations above 1200 μM led to a reduction in cell viability (*p* = 0.006), whereas exposure to 400, 600, or 800 μM HDCA enhanced cell viability (*p* < 0.01; Table S3). Under H_2_O_2_-induced oxidative stress, the highest cell viability was observed following treatment with 600 μM HDCA. Compared with the H_2_O_2_ group, cell viability was significantly increased in the 600 μM HDCA + H_2_O_2_ group (*p* < 0.001; Table S4). When the concentration of 7-ketoLCA exceeded 2000 μM, cell viability was significantly reduced (*p* < 0.001), whereas treatment with 500 or 1000 μM 7-ketoLCA significantly increased cell viability (*p* < 0.01; Table S5). Under H_2_O_2_-induced oxidative stress, treatment with 500 μM 7-ketoLCA resulted in the highest cell viability. Compared with the H_2_O_2_ group, cell viability was significantly increased in the 500 μM 7-ketoLCA + H_2_O_2_ group (*p* < 0.001; Table S6). When the concentration of CDCA exceeded 200 μM, cell viability was significantly decreased (*p* = 0.008). By contrast, treatment with 50 μM CDCA significantly increased cell viability (*p* = 0.003; Table S7). Under H_2_O_2_-induced oxidative stress, the highest cell viability was observed following treatment with 75 μM CDCA. Compared with the H_2_O_2_ group, cell viability was significantly increased in the 75 μM CDCA + H_2_O_2_ group (*p* < 0.001; Table S8). Accordingly, 600 μM HDCA, 500 μM 7-ketoLCA, and 75 μM CDCA were selected as the optimal concentrations for subsequent experiments using the H_2_O_2_-induced oxidative stress model (Figure 10A).

### 3.10. Effects of HDCA, 7-ketoLCA, and CDCA on the Expression of Intestinal Barrier-Related Proteins and Genes in IPEC-J2 Cells

As presented in Figure 10, exposure to H_2_O_2_ reduced the protein abundance of the tight junction proteins ZO-1, Occludin, and Claudin-1 relative to the Control group (*p* < 0.05). Their corresponding mRNA levels were likewise decreased following H_2_O_2_ treatment (*p* < 0.01). Compared with H_2_O_2_ exposure alone, HDCA supplementation restored the protein abundance of ZO-1 and Occludin (*p* < 0.05), while Claudin-1 protein abundance remained unchanged (*p* = 0.34; Figure 10B,C). The transcriptional responses of ZO-1 and Occludin generally paralleled their protein profiles. In contrast, Claudin-1 mRNA abundance was further reduced in the HDCA + H_2_O_2_ group (*p* = 0.007; Figure 10D). Administration of 7-ketoLCA under H_2_O_2_-induced oxidative stress enhanced Occludin protein abundance compared with the H_2_O_2_ group (*p* = 0.04), whereas ZO-1 and Claudin-1 protein abundance showed no detectable changes (*p* > 0.05; Figure 10E,F). Comparable trends were observed for the mRNA expression of ZO-1, Occludin, and Claudin-1 (Figure 10G). By contrast, CDCA treatment markedly enhanced both the protein and mRNA abundance of ZO-1, Occludin, and Claudin-1 relative to H_2_O_2_ treatment alone (*p* < 0.01; Figure 10H–J).

### 3.11. HDCA, 7-ketoLCA, and CDCA Enhance the Antioxidant Capacity of IPEC-J2 Cells by Activating the Nrf2/Keap1 Signaling Pathway

**Figure 11:**
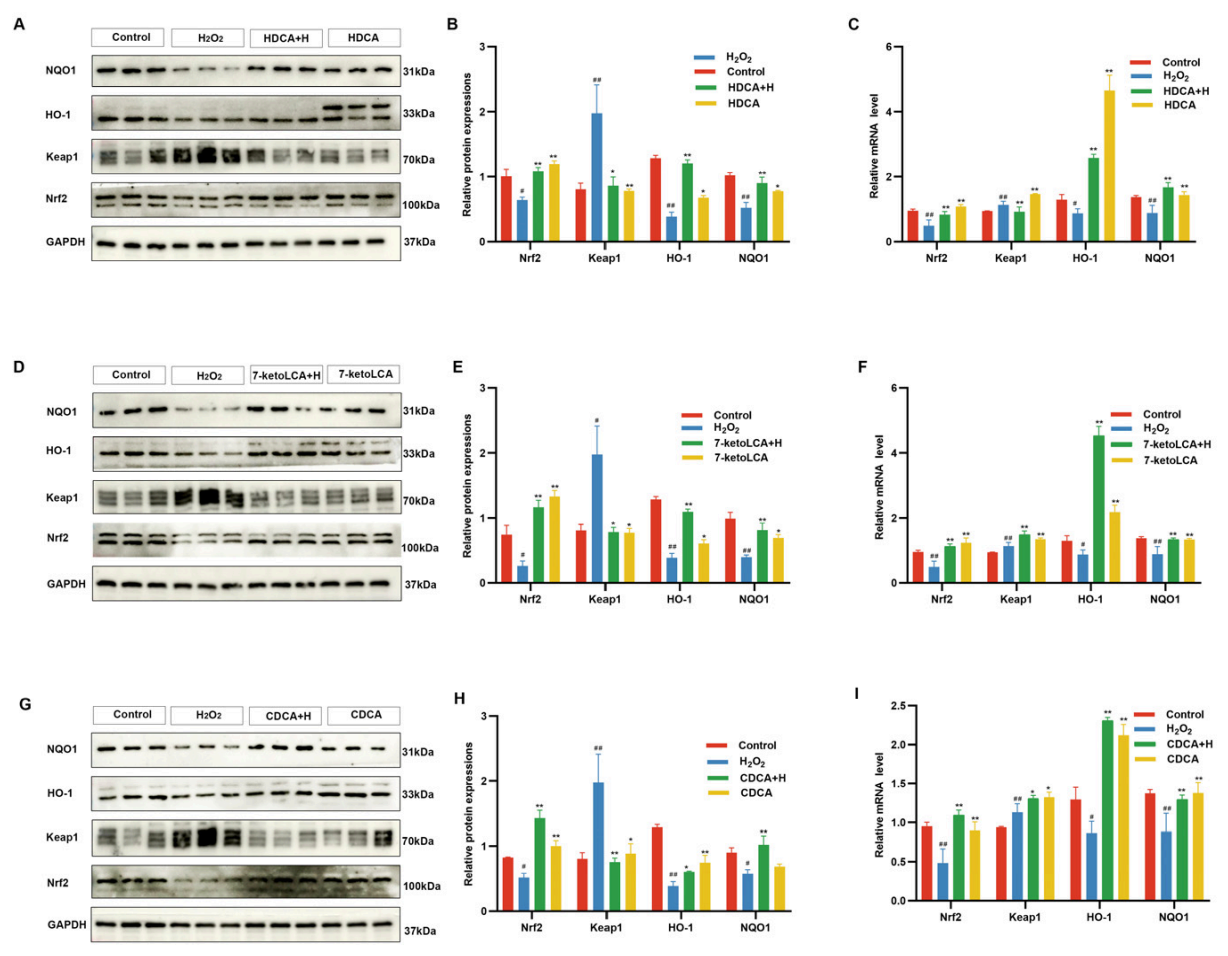
Effects of HDCA, 7-ketoLCA, and CDCA on the protein and gene expression of Nrf2/Keap1 signaling pathway-related molecules in IPEC-J2 cells (*n* = 3). (**A**,**B**) Effects of 600 μM HDCA on the protein expression levels of Nrf2/Keap1 pathway-related molecules in IPEC-J2 cells. (**C**) Effects of 600 μM HDCA on the mRNA expression levels of Nrf2/Keap1 pathway-related molecules in IPEC-J2 cells. (**D**,**E**) Effects of 500 μM 7-ketoLCA on the protein expression levels of Nrf2/Keap1 pathway-related molecules in IPEC-J2 cells. (**F**) Effects of 500 μM 7-ketoLCA on the mRNA expression levels of Nrf2/Keap1 pathway-related molecules in IPEC-J2 cells. (**G**,**H**) Effects of 75 μM CDCA on the protein expression levels of Nrf2/Keap1 pathway-related molecules in IPEC-J2 cells. (**I**) Effects of 75 μM CDCA on the mRNA expression levels of Nrf2/Keap1 pathway-related molecules in IPEC-J2 cells. # *p* < 0.05 and ## *p* < 0.01 compared with the Control group; * *p* < 0.05 and ** *p* < 0.01 compared with the H_2_O_2_ group.

As shown in Figure 11, compared with the Control group, the relative protein expression levels of Nrf2, HO-1, and NQO1 were significantly decreased in the H_2_O_2_ group (*p* < 0.05), whereas Keap1 protein expression was significantly increased (*p* < 0.05). Consistent changes were observed in the relative mRNA expression levels of Nrf2, HO-1, NQO1, and Keap1. Compared with the H_2_O_2_ group, the relative protein expression levels of Nrf2, HO-1, and NQO1 were significantly increased in the HDCA + H_2_O_2_, 7-ketoLCA + H_2_O_2_, and CDCA + H_2_O_2_ groups (*p* < 0.01), whereas Keap1 protein expression was significantly decreased (*p* < 0.05). The relative mRNA expression patterns of Nrf2, HO-1, and NQO1 were consistent with their corresponding protein expression patterns. Notably, compared with the H_2_O_2_ group, the relative mRNA expression level of Keap1 was significantly increased in the 7-ketoLCA + H_2_O_2_ and CDCA + H_2_O_2_ groups (*p* < 0.05), despite the reduction in Keap1 protein expression. Collectively, these findings indicate that HDCA, 7-ketoLCA, and CDCA enhance the antioxidant defense capacity of IPEC-J2 cells exposed to H_2_O_2_-induced oxidative stress.

### 3.12. Effects of HDCA, 7-ketoLCA, and CDCA on the Expression of Key Genes Involved in Bile Acid Metabolism in IPEC-J2 Cells

**Figure 12:**
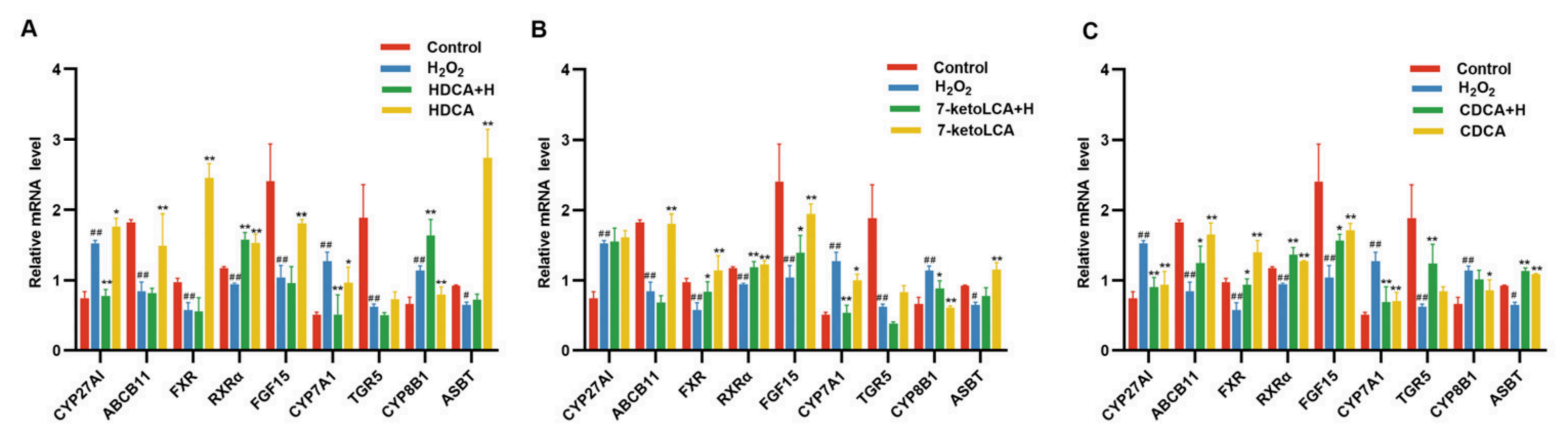
Effects of HDCA, 7-ketoLCA, and CDCA on the expression of key genes involved in bile acid metabolism in IPEC-J2 cells (*n* = 3). (**A**) Effects of 600 μM HDCA on the mRNA expression levels of key molecules involved in bile acid metabolism in IPEC-J2 cells. (**B**) Effects of 500 μM 7-ketoLCA on the mRNA expression levels of key molecules involved in bile acid metabolism in IPEC-J2 cells. (**C**) Effects of 75 μM CDCA on the mRNA expression levels of key molecules involved in bile acid metabolism in IPEC-J2 cells. # *p* < 0.05 and ## *p* < 0.01 compared with the Control group; * *p* < 0.05 and ** *p* < 0.01 compared with the H_2_O_2_ group.

Figure 12 shows that H_2_O_2_ exposure increased the mRNA abundance of the bile acid-synthesizing enzymes CYP27A1, CYP7A1, and CYP8B1 relative to the Control group (*p* < 0.01). Conversely, the transcript levels of the bile acid transporters ASBT and ABCB11 and the nuclear receptors FXR and RXRα were reduced (*p* < 0.05), while those of the membrane receptor TGR5 and the feedback regulator FGF15 showed a more pronounced decline (*p* < 0.01). Relative to H_2_O_2_ treatment alone, HDCA lowered the transcript abundance of CYP27A1 and CYP7A1 (*p* < 0.01), but elevated CYP8B1 and RXRα mRNA levels (*p* < 0.01). No detectable changes were found for ASBT, ABCB11, FXR, TGR5, or FGF15 following HDCA treatment (*p* > 0.05; Figure 12A). Compared with the H_2_O_2_ group, treatment with 7-ketoLCA had no significant effect on the relative mRNA expression levels of CYP27A1, ASBT, ABCB11, or TGR5. However, the relative mRNA expression levels of CYP7A1 and CYP8B1 were significantly decreased (*p* < 0.05), whereas those of FXR, RXRα, and FGF15 were significantly increased (*p* < 0.05; Figure 12B). CDCA treatment reduced CYP27A1 and CYP7A1 mRNA abundance relative to the H_2_O_2_ group (*p* < 0.01). Although CYP8B1 showed a downward trend, the difference between the two groups was not statistically significant. Conversely, CDCA enhanced the transcript levels of ASBT, ABCB11, FXR, RXRα, TGR5, and FGF15 (*p* < 0.05; Figure 12C). Overall, the RT-qPCR data indicated that HDCA and 7-ketoLCA modulated only selected genes associated with bile acid metabolism in IPEC-J2 cells, whereas CDCA influenced nearly all of the examined genes within this pathway. Furthermore, the transcriptional response induced by CDCA generally paralleled the mRNA and protein expression patterns of key bile acid metabolism-related molecules detected in the jejunal tissues of CAG-treated weaned piglets.

### 3.13. Effects of CDCA on the Protein Expression of Key Molecules Involved in Bile Acid Metabolism in IPEC-J2 Cells

**Figure 13:**
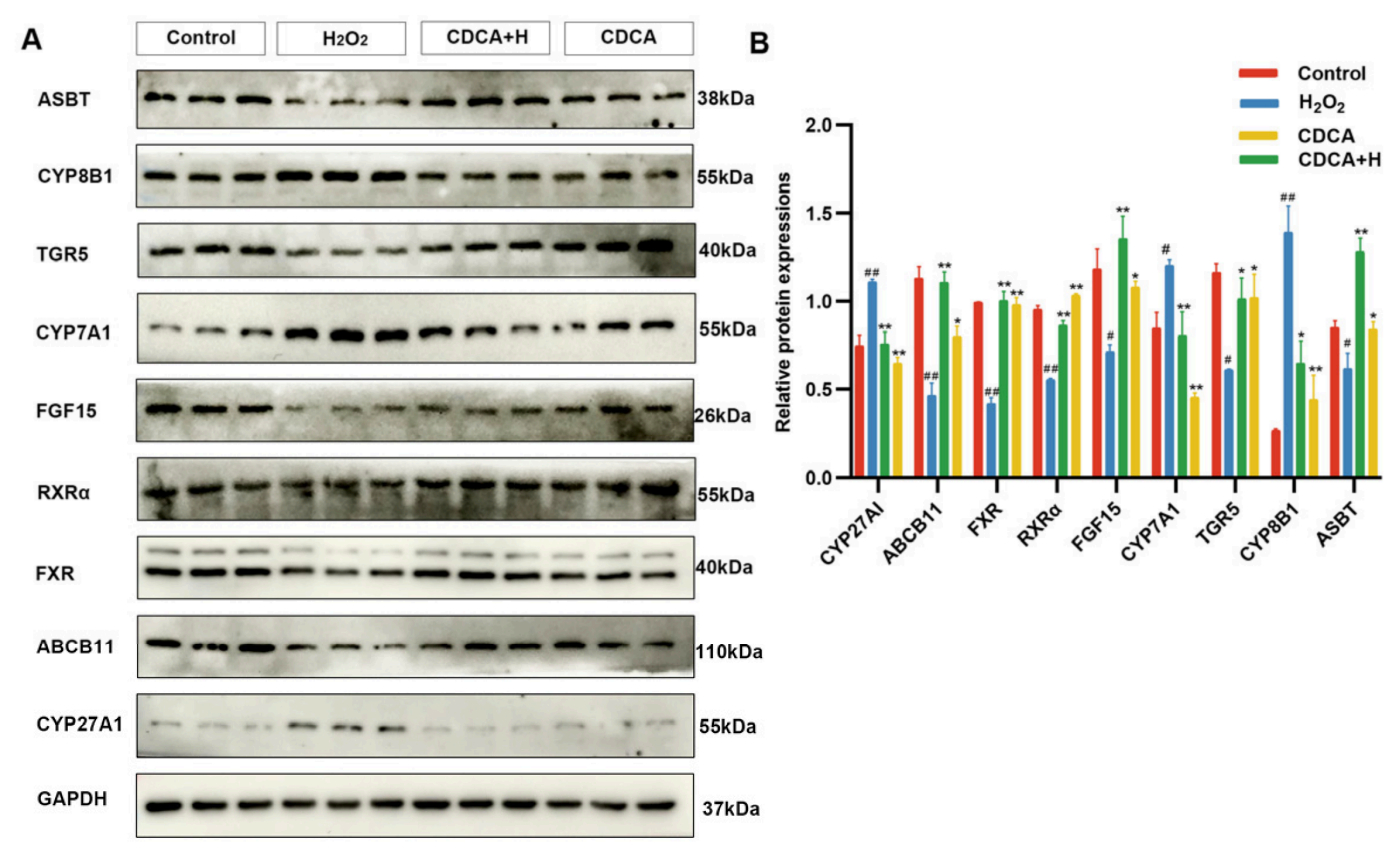
Effects of CDCA on the protein expression of key molecules involved in bile acid metabolism in IPEC-J2 cells (*n* = 3). (A) Representative Western blot bands; (B) Quantitative analysis of protein expression. # *p* < 0.05 and ## *p* < 0.01 compared with the Control group; * *p* < 0.05 and ** *p* < 0.01 compared with the H_2_O_2_ group.

Figure 13 shows that H_2_O_2_ exposure increased the protein abundance of the bile acid-synthesizing enzymes CYP27A1, CYP7A1, and CYP8B1 relative to the Control group (*p* < 0.05). Conversely, the protein levels of the bile acid transporters ASBT and ABCB11, the nuclear receptors FXR and RXRα, the membrane receptor TGR5, and the feedback regulator FGF15 were reduced following H_2_O_2_ treatment (*p* < 0.05). Compared with the H_2_O_2_ group, CDCA treatment lowered the protein abundance of CYP27A1, CYP7A1, and CYP8B1, while enhancing that of ASBT, ABCB11, FXR, RXRα, TGR5, and FGF15 (*p* < 0.05). These protein-level responses were generally consistent with the corresponding transcriptional trends observed in CDCA-treated IPEC-J2 cells.

## 4. Discussion

Weaning stress induces intestinal oxidative damage and compromises intestinal barrier integrity, thereby facilitating the translocation of endotoxins and pathogenic microorganisms through the portal circulation to the liver. This process subsequently triggers hepatic inflammation and oxidative injury, ultimately establishing a vicious cycle of “leaky gut–liver injury” [34]. Phytochemical characterization of ShenQiGan Extract identified several representative bioactive constituents, including calycosin-7-glucoside, lobetyolin, liquiritin, ononin, calycosin, liquiritigenin, and formononetin [33]. Among these compounds, flavonoid-related constituents such as calycosin, liquiritin, liquiritigenin, and formononetin have been associated with antioxidant and anti-inflammatory activities in previous studies. Together with the abundant polysaccharide and flavonoid fractions detected in the extract, these constituents may contribute to the attenuation of intestinal oxidative stress observed in the present study. Their combined biological activities may also be relevant to the regulation of the Nrf2/Keap1 signaling pathway and the subsequent improvement in intestinal redox homeostasis.

In the present study, CAG not only restored intestinal villus architecture and goblet cell abundance but also markedly alleviated hepatic histopathological lesions, suggesting that its hepatoprotective effect may be mediated, at least in part, by repairing the intestinal barrier and reducing the portal influx of gut-derived endotoxins. This coordinated protection of the gut and liver distinguishes the present findings from previous studies on plant-derived extracts, most of which have focused primarily on intestinal morphology [35,36]. Notably, improvements in serum antioxidant indices alone are insufficient to fully account for the concurrent protective effects of CAG on the intestine and liver. These effects are likely governed by upstream alterations in the gut microbiota, microbial metabolites, and associated signaling pathways. Apple polyphenols have been reported to activate the Nrf2/Keap1 signaling pathway, increase the abundance of beneficial bacteria, and reduce the population of Escherichia coli, thereby enhancing intestinal antioxidant capacity and improving epithelial barrier function. Treatment of IPEC-J2 cells with the Nrf2-specific inhibitor ML385 further confirmed the pivotal role of the Nrf2/Keap1 pathway in regulating intestinal antioxidant defenses and barrier integrity [16]. Wedelolactone has been reported to mitigate inflammatory responses and oxidative damage by reshaping bile acid metabolism and modulating the NF-κB/Nrf2 signaling network. Evidence from FXR-deficient mouse models further suggests that its hepatoprotective action against bile acid-associated injury relies on FXR activation. Through this mechanism, wedelolactone influences bile acid transport and helps re-establish enterohepatic bile acid homeostasis via the FXR–bile acid–NF-κB/Nrf2 regulatory axis [37]. Taken together, these observations support a multilevel regulatory network linking intestinal microbiota, microbial metabolites, bile acid homeostasis, and intracellular signaling, thereby offering a broader mechanistic framework for understanding oxidative stress regulation within the gut–liver axis.

A bidirectional regulatory relationship exists between the gut microbiota and bile acid metabolism. The gut microbiota catalyzes the deconjugation and biotransformation of bile acids through enzymatic systems such as bile salt hydrolases (BSHs) and hydroxysteroid dehydrogenases (HSDHs), whereas bile acids, in turn, shape the microbial community through their antimicrobial properties [22,25]. In the present study, CAG markedly enriched Lactobacillus and Limosilactobacillus, both of which contain bacterial taxa with potential BSH- and HSDH-associated functions. Concurrently, the fecal concentration of hyodeoxycholic acid (HDCA), together with the serum concentrations of HDCA, 7-ketolithocholic acid (7-ketoLCA), and chenodeoxycholic acid (CDCA), was significantly increased. Moreover, these bacterial genera were positively correlated with the altered bile acid profiles. Maternal inulin supplementation has been reported to alleviate intestinal oxidative stress in piglets by increasing the abundances of Ruminococcus and Christensenellaceae_R-7_group, elevating the levels of lithocholic acid (LCA), ursodeoxycholic acid (UDCA), and HDCA, and activating the FXR/TGR5/Nrf2 signaling pathway. Although that study verified the antioxidant effects of UDCA, LCA, and HDCA in IPEC-J2 cells, it did not further distinguish the specific biological effects of the individual bile acids [36]. Building on these findings, the present study systematically compared the regulatory effects of three differential bile acids and, to our knowledge, identified CDCA as a central metabolic hub within the bile acid network for the first time. Dietary pectin supplementation has also been shown to increase the relative abundance of Lactobacillus and the concentration of HDCA in weaned piglets; however, that study was limited to an associative analysis of the gut microbiota and bile acid profiles [38]. It should be emphasized that, although correlation analysis establishes statistical associations between microbial taxa and bile acids, the causal relationships between them require further validation through functional experiments. In a human colorectal cancer model, Bai et al. demonstrated that ginsenoside Rh4 directly promoted the conversion of CDCA to UDCA by modulating the 7α-HSDH activity of Akkermansia muciniphila [28]. This finding provides direct evidence that the gut microbiota can actively reshape the host bile acid pool. Compared with that study, the present work extends the microbiota–bile acid relationship from a disease model to a weaning-stress model and systematically characterizes the process by which CAG remodels this axis under physiological stress conditions. Collectively, these findings indicate that the gut microbiota serves as a critical intermediary in the CAG-mediated regulation of bile acid metabolism and that microbial dysbiosis may represent an important contributor to disrupted bile acid homeostasis in weaned piglets [39,40].

CAG suppressed the expression of bile acid biosynthetic enzymes while enhancing the expression of bile acid transporters and receptors. This regulatory pattern is consistent with the molecular changes reported in studies investigating the ameliorative effects of 5-aminosalicylic acid on murine colitis [41]. Compared with previous studies, the present study provides a substantially more comprehensive evaluation of the bile acid metabolic network, whereas most existing investigations have focused only on a limited number of signaling molecules, such as FXR and TGR5 [42,43]. Ursodeoxycholic acid (UDCA) has been shown to restore intestinal homeostasis and promote the colonization of Akkermansia muciniphila, thereby exerting protective effects on the intestine [44]. Similarly, dietary supplementation with hyodeoxycholic acid (HDCA) has been reported to reshape bile acid metabolism in piglets, enrich gut microbial taxa involved in bile acid biotransformation, and alter the composition of the bile acid pool, ultimately suppressing intestinal epithelial cell proliferation through the FXR-PI3K/AKT signaling pathway [45]. Through this microbiota-enzyme-bile acid regulatory axis, CAG coordinated the expression of key molecules involved in bile acid metabolism.

HDCA and 7-keto-lithocholic acid (7-keto-LCA) are secondary bile acids, whereas chenodeoxycholic acid (CDCA) is a primary bile acid. CDCA is regarded as the most potent endogenous agonist of the farnesoid X receptor (FXR). Upon FXR activation, CDCA suppresses CYP7A1 expression and therefore possesses an intrinsic advantage in initiating downstream signaling cascades [46,47]. In contrast, 7-keto-LCA and HDCA exhibit only weak FXR-activating activity and may even function as FXR antagonists. Their hepatoprotective effects are primarily mediated through activation of the downstream Nrf2 antioxidant pathway, suggesting that they predominantly exert terminal antioxidant effects rather than initiating FXR-dependent signaling [48]. In a weaned piglet model, hydroxytyrosol partially restored the oxidative stress-induced reduction in primary bile acid levels. Further correlation analysis revealed positive relationships between these bile acids and the expression of Nrf2 as well as its downstream genes [24]. In addition, Zhang et al. demonstrated that tauroursodeoxycholic acid (TUDCA) can interfere with Keap1-Nrf2 binding, thereby promoting the accumulation of Nrf2 in the nucleus and enhancing the expression of antioxidant-responsive genes [49]. These observations provide mechanistic support for a direct functional link between bile acid signaling and Nrf2-mediated antioxidant defense.

The Nrf2/Keap1 pathway plays a pivotal role in maintaining cellular redox homeostasis under oxidative stress, and abnormalities in this regulatory mechanism have been implicated in the development of inflammatory bowel disease [50,51,52]. Previous evidence indicates that trehalose enhances Nrf2-mediated antioxidant responses by facilitating the accumulation of Nrf2 in the nucleus and upregulating downstream antioxidant proteins, particularly heme oxygenase-1 (HO-1) and NAD(P)H quinone oxidoreductase 1 (NQO1). Through these effects, trehalose strengthens cellular resistance to oxidative injury [53]. In an in vitro study using IPEC-J2 cells, Lu et al. demonstrated that hyodeoxycholic acid (HDCA) alleviated intestinal oxidative stress through modulation of the Nrf2/Keap1 signaling pathway [36]. Plant-derived squalene has also been shown to increase the expression of the tight junction proteins ZO-1, occludin, and claudin-3 in piglets subjected to oxidative stress, thereby mitigating oxidative injury and preserving intestinal barrier integrity [54]. Similarly, hydroxytyrosol regulates bile acid metabolism and activates Nrf2 and its downstream antioxidant gene HO-1, subsequently increasing the expression of intestinal barrier-associated proteins, including ZO-1, occludin, MUC2, and claudin-1 [24]. These findings highlight the pivotal role of bile acids as signaling mediators linking antioxidant defense to the maintenance of intestinal barrier homeostasis.

To our knowledge, this study is the first to employ weaned piglets as an experimental model to establish a “gut microbiota–bile acid metabolism–oxidative stress” axis and to systematically elucidate the molecular mechanisms by which bile acids, as key microbial-derived signaling metabolites, regulate intestinal redox homeostasis. These findings extend the current understanding of the regulatory mechanisms underlying weaning stress-induced intestinal injury. Moving beyond the conventional focus on individual antioxidant pathways, this study further delineates the molecular targets through which CAG alleviates weaning stress-associated intestinal injury from the perspective of gut microbiota–bile acid metabolic interactions, thereby providing comprehensive experimental evidence and a theoretical basis for the development of natural bioactive compounds as environmentally sustainable alternatives to antibiotic feed additives. Nevertheless, several limitations should be acknowledged. Although PAS staining indicated that CAG supplementation increased goblet cell abundance and mucus production in intestinal tissues, and the IPEC-J2 model demonstrated protective effects of selected bile acids on epithelial barrier function, the present study did not directly determine cell-type-specific responses to individual bile acids. Future studies using cell-specific approaches are needed to clarify whether CAG-derived bile acids differentially regulate goblet cells and absorptive epithelial cells. In addition, although CAG and its associated bile acids improved antioxidant responses through Nrf2/Keap1 signaling, mitochondrial function was not directly assessed in the present study and requires further investigation. Future studies should evaluate mitochondrial homeostasis-related parameters to further elucidate the mechanisms underlying CAG-mediated antioxidant protection. Furthermore, although CAG supplementation improved hepatic histopathological alterations and functional indicators, hepatic molecular mechanisms were not comprehensively characterized in the present study. Further studies focusing on liver-specific signaling pathways, oxidative stress responses, and metabolic regulation are needed to better understand the hepatoprotective effects of CAG. Although GAPDH has been widely used as an internal reference gene in porcine intestinal tissues and IPEC-J2 cells, the stability of a single housekeeping gene may vary under different experimental conditions. Therefore, future studies should validate multiple reference genes and use their geometric mean for normalization to further improve the accuracy and reliability of qPCR analysis. Future studies will also validate the roles of key functional bacteria, particularly Lactobacillus, in weaned piglet models; establish physiologically relevant microenvironments that mimic the synergistic actions of multiple bile acids in vivo; and employ pharmacological inhibitors and gene knockout models to verify the direct functional coupling between FXR and Nrf2. These investigations will further refine the molecular framework underlying CAG-mediated regulation of intestinal oxidative stress.

## 5. Conclusions

Collectively, the findings from the in vivo and in vitro experiments demonstrate that CAG alleviates intestinal oxidative stress in weaned piglets by remodeling the gut microbial community and promoting the enrichment of functional bile acids. These alterations sequentially activate FXR/TGR5 signaling and the Nrf2/Keap1 antioxidant pathway, ultimately restoring intestinal mucosal barrier integrity. Among the altered bile acids, chenodeoxycholic acid (CDCA) acts as a pivotal molecular hub that functionally links bile acid metabolic signaling to the Nrf2/Keap1-mediated antioxidant response. This study provides systematic theoretical and experimental evidence supporting the development of CAG as a sustainable, antibiotic-free feed additive. More importantly, it proposes a gut microbiota–bile acid-Nrf2/Keap1 antioxidant axis as a key regulatory mechanism governing intestinal health in weaned piglets, thereby identifying a well-defined mechanistic target for the precise prevention and intervention of weaning-associated intestinal oxidative stress.

## Figures and Tables

**Table 1:** Major phytochemical constituents of ShenQiGan Extract previously identified and quantified by HPLC. Data from Guo et al. [33].

Phytochemical Constituent	Content, μg/mL
Calycosin-7-glucoside	19.8
Lobetyolin	23.1
Liquiritin	86.8
Ononin	14.2
Calycosin	16.4
Liquiritigenin	35.7
Formononetin	17.2

**Table 2:** Serum antioxidant status of weaned piglets following dietary CAG supplementation.

Item	Control	LCAG	MCAG	HCAG	SEM	*p*-Value
GSH-Px (U/mL)	1054.34	1251.73 *	1319.56 **	1190.86	59.65	0.002
MDA (nmol/mL)	2.32	1.64 **	1.39 **	1.33 **	0.146	0.000
SOD (U/mL)	23.04	25.11 **	24.04	25.28 **	0.557	0.002
CAT (U/mL)	4.19	4.88	4.50	5.81 **	0.355	0.004

GSH-Px = glutathione peroxidase; MDA = malondialdehyde; CAT = catalase; SOD = superoxide dismutase. Control, piglets fed the basal diet; LCAG, piglets fed the basal diet supplemented with 0.1% CAG; MCAG, piglets fed the basal diet supplemented with 0.5% CAG; HCAG, piglets fed the basal diet supplemented with 1.0% CAG. * *p* < 0.05 and ** *p* < 0.01 compared with the Control group.

**Table 3:** Effects of different concentrations of H_2_O_2_ on the viability of IPEC-J2 cells.

Items	Concentration of H_2_O_2_, μmol/L								SEM	*p*-Value
	0	200	400	600	800	1000	1200	1500		
Cell viability, %	100.00	97.82	92.73 **	77.78 **	68.16 **	64.77 **	62.93 **	11.42 **	1.47	0.000

H_2_O_2_ = hydrogen peroxide. ** *p* < 0.01 compared with the Control group (0 μmol/L H_2_O_2_).

## Data Availability

The sequencing data have been deposited in the NCBI Sequence Read Archive (SRA) and are publicly available under the accession number PRJNA1527609. The data can be accessed at the following link: https://www.ncbi.nlm.nih.gov/sra/?term=PRJNA1527609 accessed on 9 September 2026.

## Supplementary Materials

The following supporting information can be downloaded at: https://www.mdpi.com/article/10.3390/antiox15101323/s1, Table S1. Primer sequences used for real-time quantitative PCR (RT-qPCR). Table S2. Effects of different concentrations of DMSO on the viability of IPEC-J2 cells. Table S3. Effects of different concentrations of HDCA on the viability of IPEC-J2 cells. Table S4. Effects of HDCA on the viability of H_2_O_2_-treated IPEC-J2 cells. Table S5. Effects of different concentrations of 7-ketoLCA on the viability of IPEC-J2 cells. Table S6. Effects of 7-ketoLCA on the viability of H_2_O_2_-treated IPEC-J2 cells. Table S7. Effects of different concentrations of CDCA on the viability of IPEC-J2 cells. Table S8. Effects of CDCA on the viability of H_2_O_2_-treated IPEC-J2 cells.

## Abbreviations

7-ketoLCA	7-ketolithocholic acid
CAG	ShenQiGan Extract
CDCA	Chenodeoxycholic acid
FXR	farnesoid X receptor
GSH-Px	glutathione peroxidase
HDCA	hyodeoxycholic acid
HO-1	heme oxygenase-1
LC-MS	liquid chromatography–tandem mass spectrometry
NQO1	NAD(P)H quinone dehydrogenase 1
ROS	reactive oxygen species
RXRα	retinoid X receptor alpha
SOD	superoxide dismutase
TGR5	Takeda G protein-coupled receptor 5
