# Supplementary material for: ShenQiGan Extract Mitigates Intestinal Oxidative Stress in Weaned Piglets Through Bile Acid Metabolism Regulation and Gut Microbiota Remodeling via the Nrf2/Keap1 Pathway
Source: Antioxidants (Basel). 2026 Oct 14;15(10):1323. doi: 10.3390/antiox15101323 (PMC13701692; doi:10.3390/antiox15101323)
Supplement: Supplementary file 1 [file antioxidants-15-01323-s001.zip › antioxidants-4585316-supplementary.pdf]

Table S1. Primer sequences used for real-time quantitative PCR (RT-qPCR).

Gene	Forward primer (5′-3′)	Reverse primer (5′-3′)
GAPDH	GTCGGAGTGAACGGATTTGGC	GGAGGTCAATGAAGGGGTCA
Nrf2	GACAGGCGAATTCCTCCCAA	GAATGTGGGCTACCTGGGAA
Keap1	ATGGCGGGGCCTCTGA	CTCAGGGGCAGAAATTGGGT
HO-1	CGAGAAGGCTTTAAGCTGGTG	CGGGTTCTCCTTGTTGTGCT
NQO1	CCTCTGGCCAATTCAGAGTGG	CAGGATCTGGATTCGGGCAT
CYP27A1	GAGGGCAAGTACCCAGTACG	ATGACTCTCCTTCCGTGGTG
ABCB11	AGCAAAAGGCTGTTTCAGATGT	CATCACTGTAAAGCACCTTGGA
FXR	TGAGGGGTGCAAAGGTTTCT	TCACACAGTTGCCCCCATTT
RXRα	CAAGTGCCTGGAACACCTCT	ATGGAAGGTAACAGGGTGGC
FGF15	AAGATGCAAGGGCAGACTCA	AGATGGTGTTTCTTGGACCAGT
CYP7A1	CCGCTTCTGATACCTGTGGA	GGTTTGCTCGGAGGAACTCA
ASBT	AGGCATATCTTTGGTCGCTCT	GCCTGTGATGGATCCAACCTTA
TGR5	CCATGCACCCCTGTTGCT	GGTGCTGTTGGGTGTCATCTT
CYP8B1	CCTCTTTCCTTTGGCCCCAT	TGGTGCTGGCTGAGTGTATC
ZO-1	CCAACCATGTCTTGAAGCAGC	TGCAGGAGTGTGGTCTTCAC
Occludin	CAGGTGCACCCTCCAGATTG	TGGACTTTCAAGAGGCCTGG
Claudin1	AGGAGGGAAGCCATTTTCATGT	TATTTAAGGACCGCCCTCTCC

Table S2. Effects of different concentrations of DMSO on the viability of IPEC-J2 cells.

Items	Concentration of DMSO（%）								SEM	*P*-value
	0.00	0.10	0.30	0.50	0.70	1.00	1.50	2.00		
Cell viability, %	100.00[a]	101.37[a]	96.29[a]	93.53[a]	94.42[a]	88.94[a]	81.07[ab]	67.09[b]	6.35	<0.001

DMSO, dimethyl sulfoxide.

[1]Within a row, means without a common superscript letter differ at $P < 0.05$, n =6.

Table S3. Effects of different concentrations of HDCA on the viability of IPEC-J2 cells

Items	Concentration of HDCA, μM						SEM	*P*-value
	0.00	400	600	800	1000	1200		
Cell viability, %	100.94[c]	117.11[ab]	118.78[a]	109.72[bc]	102.44[c]	89.49[d]	3.00	<0.001

HDCA, hyodeoxycholic acid .

[1]Within a row, means without a common superscript letter differ at $P < 0.05$, n =6.

Table S4. Effects of HDCA on the viability of H_2O_2-treated IPEC-J2 cells.

Item	Blank	H_2O_2	Concentration of HDCA, μM			SEM	*P*-value
			400	600	800		
Cell viability, %	100.00[a]	68.89[c]	75.86[b]	78.40[b]	55.81[d]	1.628	<0.001

HDCA, hyodeoxycholic acid; H_2O_2, hydrogen peroxide.

[1]Within a row, means without a common superscript letter differ at $P < 0.05$, n =6.

Table S5. Effects of different concentrations of 7-ketoLCA on the viability of IPEC-J2 cells

Items	Concentration of 7-ketoLCA, μM						SEM	*P*-value
	0.00	500	1000	1500	2000	2500		
Cell viability, %	100.00^{b}	121.52^{a}	104.41^{b}	97.47^{b}	75.04^{c}	65.01^{d}	2.07	<0.00

7-K-LCA, 7-ketolithocholic acid,

[1]Within a row, means without a common superscript letter differ at $P < 0.05$, n =6.

Table S6. Effects of 7-ketoLCA on the viability of H_2O_2-treated IPEC-J2 cells.

Item	Blank	H_2O_2	Concentration of 7-ketoLCA, μM			SEM	*P*-value
			500	1000	1500		
Cell viability, %	100.75^{a}	74.11^{c}	84.72^{b}	66.39^{d}	33.75^{e}	1.79	<0.001

7-K-LCA, 7-ketolithocholic acid; H_2O_2, hydrogen peroxide.

[1]Within a row, means without a common superscript letter differ at $P < 0.05$, n =6.

Table S7. Effects of different concentrations of CDCA on the viability of IPEC-J2 cells

Items	Concentration of CDCA, μM					SEM	*P*-value
	0.00	50	100	200	400		
Cell viability, %	100.00^{b}	111.43^{a}	101.66^{b}	88.89^{c}	41.80^{d}	2.95	<0.001

CDCA, chenodeoxycholic acid.

[1]Within a row, means without a common superscript letter differ at $P < 0.05$, n =6.

Table S8. Effects of CDCA on the viability of H_2O_2-treated IPEC-J2 cells.

Item	Blank	H_2O_2	Concentration of CDCA, μM			SEM	*P*-value
			50	75	100		
Cell viability, %	100.62[a]	70.15[d]	85.73[c]	93.40[b]	67.89[d]	1.77	<0.001

CDCA, chenodeoxycholic acid; H_2O_2, hydrogen peroxide.

[1]Within a row, means without a common superscript letter differ at $P < 0.05$, $n = 6$.
